# Security Engineering of Patient-Centered Health Care Information Systems in Peer-to-Peer Environments: Systematic Review

**DOI:** 10.2196/24460

**Published:** 2021-11-15

**Authors:** Imrana Abdullahi Yari, Tobias Dehling, Felix Kluge, Juergen Geck, Ali Sunyaev, Bjoern Eskofier

**Affiliations:** 1 Department of Artificial Intelligence in Biomedical Engineering, Machine Learning and Data Analytics Lab Friedrich-Alexander University Erlangen-Nuremberg Erlangen Germany; 2 Institute of Applied Informatics and Formal Description Methods Karlsruhe Institute of Technology Karlsruhe Germany; 3 KASTEL Security Research Labs Karlsruhe Germany; 4 REFINIO GmbH Rohr Germany

**Keywords:** patient-centered, health care, information infrastructures, decentralization, mobile health, peer-to-peer, COVID-19 proximity trackers, edge computing, security, vulnerabilities, attacks, threats, mobile phone

## Abstract

**Background:**

Patient-centered health care information systems (PHSs) enable patients to take control and become knowledgeable about their own health, preferably in a secure environment. Current and emerging PHSs use either a centralized database, peer-to-peer (P2P) technology, or distributed ledger technology for PHS deployment. The evolving COVID-19 decentralized Bluetooth-based tracing systems are examples of disease-centric P2P PHSs. Although using P2P technology for the provision of PHSs can be flexible, scalable, resilient to a single point of failure, and inexpensive for patients, the use of health information on P2P networks poses major security issues as users must manage information security largely by themselves.

**Objective:**

This study aims to identify the inherent security issues for PHS deployment in P2P networks and how they can be overcome. In addition, this study reviews different P2P architectures and proposes a suitable architecture for P2P PHS deployment.

**Methods:**

A systematic literature review was conducted following PRISMA (Preferred Reporting Items for Systematic Reviews and Meta-Analyses) reporting guidelines. Thematic analysis was used for data analysis. We searched the following databases: IEEE Digital Library, PubMed, Science Direct, ACM Digital Library, Scopus, and Semantic Scholar. The search was conducted on articles published between 2008 and 2020. The Common Vulnerability Scoring System was used as a guide for rating security issues.

**Results:**

Our findings are consolidated into 8 key security issues associated with PHS implementation and deployment on P2P networks and 7 factors promoting them. Moreover, we propose a suitable architecture for P2P PHSs and guidelines for the provision of PHSs while maintaining information security.

**Conclusions:**

Despite the clear advantages of P2P PHSs, the absence of centralized controls and inconsistent views of the network on some P2P systems have profound adverse impacts in terms of security. The security issues identified in this study need to be addressed to increase patients’ intention to use PHSs on P2P networks by making them safe to use.

## Introduction

### Motivation

Patients require access to their health information with the same ease as with other web-based activities such as banking or shopping; however, patients are often only one part of the current health care processes and not the focus of attention [1]. Such limitations of traditional health care processes, widespread individual adoption of digital systems, and advancements in health care practice create a growing demand for patient-centered health care information systems (PHSs). PHSs are scalable information systems that leverage information technology to support patients in managing and taking an active role in their own health [1,2]. PHSs are not designed to replace traditional health care information systems, such as electronic health records, but rather to complement them [3] by offering additional functionalities, such as translation of clinical information into layman’s terms [4], provision of information on medications a patient is taking [2,5], or provision of vetted information to support self-administered interventions (eg, reduce weight or quit smoking) [4].

The diversity and flexibility of PHSs enable them to provide any functionality that patients find helpful [2], including maintaining personal health records (PHRs) [6], tracking mental wellness [7], subscribing to risk prediction services for chronic diseases [6,8], and calculating pregnancy due dates [9]. Patients are willing to use PHSs, as revealed in a survey of 800 American patients in which 80% of the patients preferred a patient-centered approach as they felt excluded in the management of their data [10]. With PHSs, patients can access their health information and share it with other stakeholders to co-ordinate their care [1]. Practitioners can make better clinical decisions based on instantaneous access to data in PHSs [11]. In patient-centered health care environments, the value for patients is increased, health care transaction costs are decreased, patients manage interactions through the appropriate release of their own data, and all health care stakeholders will be encouraged to collaborate with patients and other stakeholders to achieve their goals [1].

Technically, PHSs can be deployed using centralized databases (eg, Health Bank [12], Microsoft HealthVault [3], and PittPHR [13]), distributed ledger technology (DLT; eg, Mint Health [14] and Medicalchain [11]), and more flexible peer-to-peer (P2P) technology (eg, OnePatient [15] and doc.ai [7]).

The detrimental effects of centralized health information technology solutions controlled by economic actors are well-known [16], for example, reluctance to innovate or the creation of data silos [16]. DLT-based PHSs, such as MedRec, which is under development at the Massachusetts Institute of Technology [17], are currently spurring the P2P and decentralization push in the health care domain. However, DLT is a specialized P2P technology that does not align well with the needs of the health care sector and the sensitivity of health information. For instance, DLT systems consume excessive computation and communication resources by requiring redundant computations to ensure a consistent state of the ledger across the network, which makes the logged transactions available to all nodes participating in the network, and they have slow processing speeds because multiple parties have to independently verify transactions and arrive at an agreement [18]. The mismatch between DLT and the needs of the health care sector has a simple cause: DLT was primarily designed as a backbone for cryptocurrencies that require one global consistent record of transactions and can thrive even in environments where trusted counterparties do not exist and might even be malicious [19]. Accordingly, DLT is a P2P technology that is too rigid for the health care context, where it is sufficient for all parties involved in the care of a patient to have a consistent view of a patient’s health status and existing trust relationships between parties (eg, the patient-physician relationship) can be leveraged. In this study, we take an information security perspective and contribute to the emergence of PHSs that come with the benefits promised by DLT PHSs, such as decentralization, patient empowerment, and interoperable health systems [18], but are implemented based on less rigid and more flexible P2P technology. We refer to such systems as P2P PHSs.

P2P PHS architectures can be based on hybrid P2P networks (eg, P2HR [20]), approaches that combine centralized and P2P architectures (eg, P2P PHR [6] or the e-toile framework in Switzerland [21]), and highly decentralized networks (eg, P2P-integrating health care enterprise [P2P IHE; 22]). Other examples of P2P PHSs, which are disease-centric, are decentralized systems for Bluetooth-based SARS-CoV-2 (or COVID-19) contact tracing, for example, Pan-European Privacy-Preserving-Proximity-Tracing (PEPP-PT) in Europe [22], Trace-Together in Singapore [23], and Stoop in Austria [24], which are used to notify people when they are near SARS-CoV-2 carriers.

In P2P PHSs, the trust and identity of individual participants do not need to be assured through technology. P2P PHSs provide PHS functionalities locally (on any patient edge device such as mobile phones, tablets, etc) under the sovereignty of individual device owners. Patients can make their health information directly available to other participants they trust without the need for any centralized or distributed nodes to facilitate the transactions. However, P2P PHSs have unique security issues because patients must manage information security for their health information largely by themselves, and even qualified professional administrators are already challenged by the task [25]. The absence of a central entity to act as a trusted computing base on P2P networks [25,26] has profound adverse consequences in terms of security that need to be addressed to reap the benefits that P2P PHSs promise to offer.

### Objectives

P2P PHSs raise challenging information security–related questions: How can reliable data backups be implemented? If credentials are lost or compromised, how can they be replaced or blocked? How well is the system protected against unauthorized access? P2P PHSs that are not DLT-based (eg, OnePatient [15] and P2P PHR [6]) are an emerging phenomenon that will become more relevant in the future as they are aligned well with large-scale efforts to re-decentralize the internet (eg, the Solid project by Tim Berners-Lee [27]) and support patients in taking ownership of their health data [1,10]. Although P2P PHSs have been under development for over a decade [21], the dedicated literature on P2P PHSs is sparse. To date, previous studies have focused on security, privacy, and end-user features on centralized and DLT-based PHSs [2,28-31] and did not address security engineering specifically for P2P PHSs, which comes with its own challenges due to a different underlying architecture. To address this gap, this study focuses on security engineering for P2P PHSs based on a systematic literature review. We aim to answer the following research question:

Research question: What are the inherent security issues for PHS deployment on P2P networks and how can they be overcome?

Security issues are defined as any action that could be used to disrupt the functionality of the P2P network or enable unauthorized users to access, modify, or delete user data [32,33], specifically, due to threats or vulnerabilities, such as malware, bugs, access control failures, or patients' inadvertent exposure of their data. To answer the research question, we aim to review existing P2P and P2P PHS architectures and their design choices, study existing PHS features, and propose a suitable architecture for PHS deployment on P2P networks. Thereafter, we aim to highlight the causes and consequences of existing security issues in P2P PHSs and evaluate them based on the identified P2P PHSs in the literature. On the basis of these P2P PHS architectures, we propose security measures for secure provision. To overcome the challenges on the path to P2P PHSs, secure safeguards must be put in place to ensure that information is securely transmitted and protected against cyberattacks [1,34]. Information security is essential for P2P PHSs and will, if appropriately implemented and addressed, increase patients' intention to use P2P PHSs [2,30].

### Theoretical Background

#### P2P PHSs and the Need for Information Security

P2P technology for the provision of PHSs can be flexible and inexpensive for users because it uses available devices at the user’s end for deployment. The characteristics of P2P systems, such as fault tolerance, security and trust, scalability, availability, self-reconfiguration, and extensibility [35,36], facilitate and suit the provision of PHSs. With millions of users worldwide, P2P systems have shown strength in providing services for sharing resources without the need for a central server, for streaming multimedia content with distributed load balancing, for volunteering of computing resources, and for telephony applications. P2P PHSs, such as OnePatient [15] and P2P PHR [6], leverage the power of P2P networks and mobile technology to store health records locally under the control of device owners, thereby increasing patient empowerment and control and simplifying the implementation of data protection principles [8,37,38]. P2P systems have better scalability because operations can be executed locally and customized for different purposes. Patients can easily manage access to their health records by using a single-hop connection (eg, Wi-Fi Direct) with other trusted parties (eg, a physician) without requiring a wireless access point or another intermediary communication network.

Factors that impact the security of centralized PHSs are the database size, the large number of potentially affected users, and the confidentiality of the stored data. The health care sector experiences more data breaches than any other sector [39]. A breach barometer in the United States reported 503 breaches for health data in 2018, affecting over 15 million patients [40]. Similarly, the almost immutable nature of data storage in blockchains makes it nearly impossible for users to erase their stored (metadata) information, which conflicts with the European General Data Protection Regulation (GDPR) [41]. Table 1 outlines the main advantages and disadvantages of P2P PHSs.

For patients to benefit from the advantages of P2P PHSs, the network needs to be robust and fault-tolerant. Information security is paramount because of the high sensitivity of medical data [30,42]. Therefore, a pertinent question is how to make P2P PHSs resilient to attacks. P2P systems communicate over the internet; therefore, they inherit the same security issues as any other networked application on the internet. The P2P architecture poses significant security issues such as index poisoning attacks [43], Sybil attacks [44], chatty peer attacks [45], or distributed denial-of-service (DDoS) attacks [46].

**Table 1:** Security advantages and disadvantages of peer-to-peer patient-centered health care information systems (P2P PHSs).

Dimension	Advantages	Disadvantages
Privacy management	Patients technically govern data. Patients can define access rights to their own PHSs.	Inconsistent views in the network allow attackers (and super users) to cheat and remain undetected.
Federated medical data	Patients keep their medical data and software on their own devices. Patients can determine the desired redundancy for their data by backing up at their end.	Patients may lose access when the device is lost, and no backup system is used by the patient.
Security	No central attack profiles.	Specific security issues other than general networked application attacks are introduced and slow deployment of security patches by users results in insecure P2P systems.
Offline capability	Data are available without a network connection, which improves infrastructure resilience. Disrupted internet connections will not stop data access.	Maintenance effort for storing large amounts of data offline can be high.
Stakeholder interaction management	All health care stakeholders requiring access to patient data have to interact with patients to achieve their goals.	Increased access control requirements for patients are hard to satisfy with current health care processes and systems due to bureaucracy and diverse levels of digitalization.

Moreover, P2P systems increase the attack surface owing to 3 disadvantages [26,47]: (1) increased chances of exposing network traffic patterns to attackers; even with encryption, the metadata can still reveal information to external attackers; (2) an inconsistent view of the network (due to a lack of global information), which affects integrity by allowing attackers to cheat and remain undetected; and (3) increased vulnerability to internal attackers due to the absence of a central entity to detect malicious insiders and govern software and security updates.

#### P2P and PHS Networks

#### Origins

**Figure 1:**
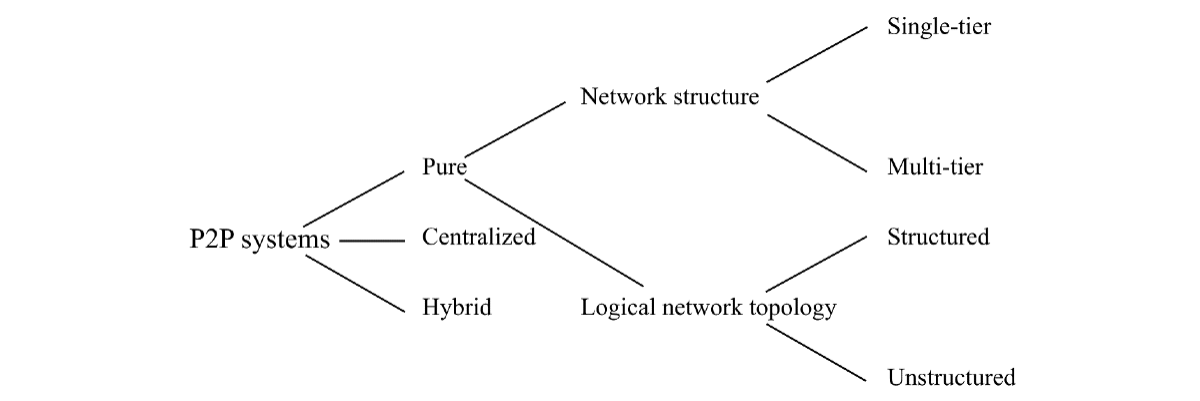
Peer-to-peer (P2P) architectures. Some P2P systems are supported by centralized servers, other P2P systems attempt to decentralize as far as possible. Between these two extremes, hybrid systems benefit from the properties of both.

The concept of P2P was introduced in 1969 in the first Request for Comments of the Internet Engineering Task Force; Request for Comments-1 denotes a *host-to-host connection* [48]. UseNet [49], a distributed messaging system, is often described as the first true implementation of a P2P network and was established in 1979. UseNet looks like a client server model from users' point of view. However, servers communicate with each other based on the concept of P2P and share content over the entire group of UseNet servers without a central entity. With the surge in popularity of P2P networks, the music and file-sharing P2P application Napster [50] was introduced in 1999, which exhibited some approaches to P2P networks known today. Later, well-known and popular P2P systems emerged, such as Gnutella, eDonkey, and BitTorrent. Within the last 2 decades, the first health information systems were deployed on P2P networks—for example, the e-toile P2P PHS framework aimed at connecting all health care stakeholders in Geneva, Switzerland [21,51]; P2HR [20]; or the PEPP-PT COVID-19 contact tracing system in Europe [22]. The features distinguishing P2P systems from centralized systems are peer and resource discovery [35]. Since there are no servers, peers (eg, patients, practitioners, or PHS providers) must rely on techniques, such as indexing and routing tables [52], to locate other peers in the network (Figure 1).

A P2P network, or system, is a type of computer network that exhibits decentralized control, autonomy, virtualization, and sharing of computing resources [47,50]. Peers participating in the network form a P2P network of nodes and are equally privileged. The network is self-organizing. Peers in the network make their resources directly available to other peers without the need for a central entity to facilitate or co-ordinate transactions [35]—for example, patients can directly exchange information with practitioners over their P2P PHSs. Peers in a P2P network can share and download resources. This is in direct contrast to traditional client-server networks in which resource-sharing and downloading are performed by distinct actors (eg, in PHRs such as Google Health or Microsoft Health Vault).

#### Centralized

**Figure 2:**
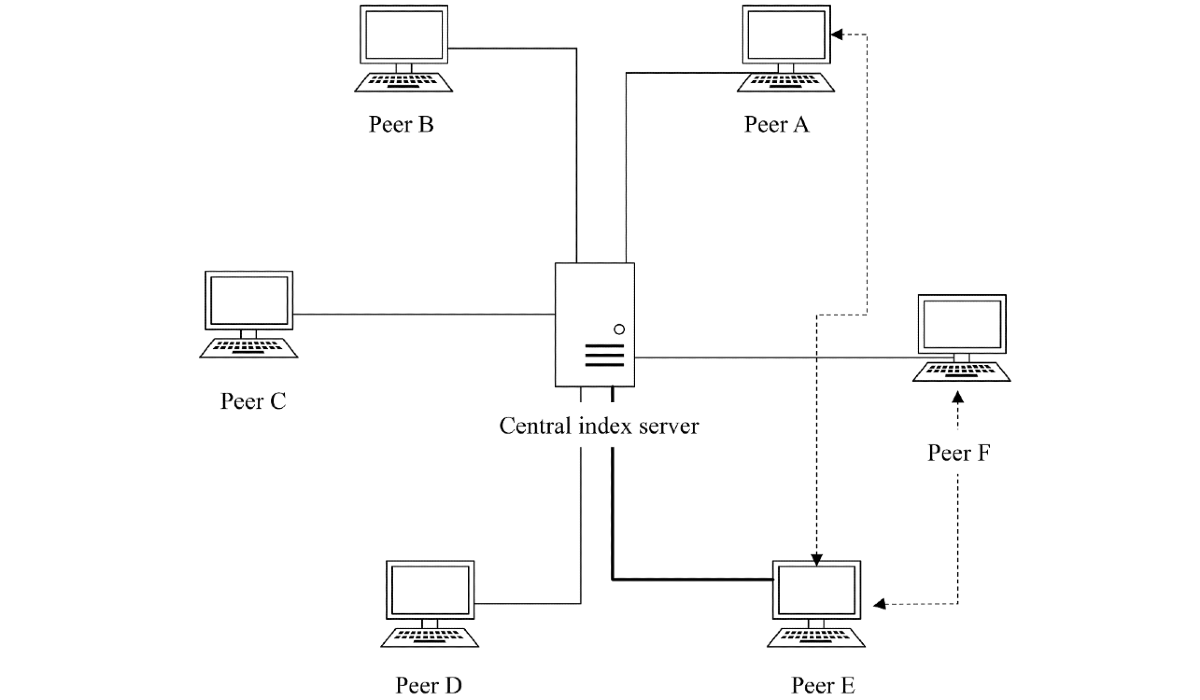
The centralized peer-to-peer (P2P) system. A peer E sends a message to the central server asking for the desired resource, the server runs a lookup and determines the peers that contain the queried resource and then sends back the result to the requesting peer E. Once peer E obtained the list (which consists of peer A and peer F), it establishes a direct connection to the peers.

Centralized P2P PHS (eg, P2P PHR [6] and e-toile framework [21]), and other centralized P2P systems (Napster, SETI@Home, and BOINC [35,50]) combine the features from client-server and decentralized architectures. One or more central servers are used to manage administration, transaction, registration, or resource discovery. To abide by data protection regulations, such as the US Federal Health Insurance Portability and Accountability Act (HIPAA) [6] or the GDPR [34,41], and related regulations, health or personal information should be stored separately from centrally managed operational data (eg, status and metadata of transactions as in P2P PHR [6] or the list of interoperable PHS providers and health care professionals and their access rights in the e-toile framework [21]). In the case of contact tracing systems such as PEPP-PT COVID-19 [22], the central server may be operated by a government or trusted entity to generate identities and contact graphs. In centralized P2P PHSs, the resources are indexed by the central server (Figure 2). Although a client-server approach is used for resource discovery, the actual communication that facilitates resource transmission is decentralized [53].

In centralized P2P PHSs, data protection and security measures based on regulations such as HIPAA [6] or GDPR [41] can be enforced and implemented but PHSs may inherit issues from centralized systems [35], such as vulnerability to insider attacks and function creep by the entity running the server; reduced tolerance to avoid single points of failure; and issues with scalability and robustness. Central servers also become more likely to cause a bottleneck when the number of peers increases.

#### Decentralized

In decentralized P2P systems, peers have equal rights and responsibilities [35,54]. This can be seen in agent-based co-ordination frameworks proposed for the exchange of electronic health records between different providers (eg, P2P IHE [6,51]) or other P2P systems (eg, BitTorrent, Gnutella, Freenet, Chord, and PAST [35,50]). Each peer shares data that may only be relevant to queries of other peers. A decentralized P2P design is a user-based infrastructure because it requires no specific additional infrastructure and depends solely on the participating users to share resources (bandwidth and storage) [26]. In a decentralized P2P system architecture, 2 further dimensions are important [35]: the *network structure* and *logical network topology* (overlay network).

The *network structure* of a P2P network can be *single-tier* or *multitier*. In a *single-tier* network (eg, Gnutella, Freenet, and PAST [35,50]), loads and functionalities are equally distributed among the nodes participating in the network. In contrast, the *multitier* network has a routing structure with hierarchical layers. An example of a P2P protocol in this category includes the Super-peer Architecture and Crescendo System [35].

The *logical network topology* can be *structured* or *unstructured*. In *unstructured* P2P networks (eg, FreeNet, Gnutella, and KaZaA [50]), which exhibit a mesh topology [26], each peer maintains the list of its neighbors to which it may forward queries. Hence, in most cases, a peer must search a large fraction of the network when looking for a desired resource in the network, as there is no precise mapping between the identifiers of resources and peers [55]. Messages are continuously propagated by neighbors in the network [26], which affects the reliability of message delivery when the network is congested. This type of P2P system can be unsuitable for PHS deployment, especially in emergency situations where a patient’s medical history (located with another remote peer) is urgently needed for medical care.

To address these problems, *structured* P2P PHSs such as P2P IHE [51] and other *structured* P2P systems (eg, Chord, Kademlia, Pastry, and CAN [35]) have emerged. In *structured* P2P systems, a mapping between peers and data exists, data placement is under the control of Distributed Hash Tables (DHTs), and each peer has to maintain routing tables. A DHT is a hash table containing a key-value lookup function, and the entire index is equally distributed among participating peers [55]. The key-value store represents only the metadata of the participating peers, for example, the mapping (id, ptr) indicates that a resource with identifier *id* is located at a peer pointed to by *ptr*. The general idea of structured P2P networks is to minimize the number of peer lookups (eg, by adopting a key-based routing strategy) to identify and locate a desired resource in the network [35]. The cost of maintaining the structured topology is high when participants arbitrarily join and leave the network.

The overall issue of decentralized P2P systems is the slow search for peers offering the desired resources in the network [35], and freedom to join or leave the network affects availability [20,56]. However, these systems do not have single points of failure and benefit from other features, such as scalability and robustness to operational errors. The lack of centralized control is a major factor contributing to routing difficulties: routing becomes more complicated with more diverse participating nodes [57], when massive peer churn is present [58] and when there is a dependence on nodes that could be malicious [59]. To remedy this, a shared memory in a distributed tuple space architecture [60], as used in the P2P PHS agent-based co-ordination framework P2P IHE [51], can be leveraged. In such an architecture, a distributed network of tuple centers is used as a co-ordination framework to facilitate interactions between various PHS providers and other health care stakeholders [51].

#### Hybrid

P2HR [20] is an example of a hybrid P2P PHS. Other P2P systems (eg, BestPeer [35], *BestPeer++* [61], or BitTorrent [62]) eventually relied on this topology. Hybrid P2P architectures were introduced to address the challenges of centralized servers in P2P networks and the time required for resource discovery in decentralized P2P networks [35,54]. They combine the advantages of both architectures [50], such as reliable resource discovery and scalability. Although there are no servers in hybrid P2P systems, peer nodes that have more resources in terms of storage, computation power, network connectivity, stability, and uptime can fulfill the role of servers and assist *common peers* with resource discovery. These nodes are referred to as *super peers*. In hybrid P2P systems, resource discovery can be performed by querying the *super peer* (in a centralized manner) or using decentralized search techniques [63]. *Common peers* form the lower layer, while *super peers* form the upper layer.

Although *super peers* share some similar properties with servers in a centralized P2P network, they are different [35]: (1) a *super peer* only acts as a manager for its subset of peers in the network—it is not as powerful as a server in centralized P2P networks that oversees the entire network. For PHSs, dividing patients into groups (eg, per hospital) ensures that patients’ data are only shared with users that require them [64]; (2) a *super peer* also participates and acts as a *common peer* and facilitates the same operations, such as resource-sharing and downloading. As an analogy, the relationship of *super peers* with *common peers* is similar to interactions between entities in human society: for instance, in a hospital, physicians keep more knowledge and connections with their patients than other personnel. As such, patients with health issues are expected to ask for help from physicians, as there is a higher probability that they are able to handle the problem.

*Super peers* can act as *federated* authorities whereby participating users can affiliate themselves with provider nodes based on extant trust relationships (eg, friendship or treatment relationships). Provider nodes are largely independent of each other; hence, there is a federation of provider nodes. Each provider is responsible for its common peers; however, individual provider nodes can collaborate to provide services. The placement of *super peers* in a privileged position enhances the availability of resources, operations, computations, and performance; however, this also raises issues regarding trust, privacy, and integrity as *super peers* regulate services. The absence of a *super peer* in the network may affect operations in the network, thereby reducing the fault tolerance of the P2P network. In terms of security, nodes operated by providers are central points of attack (at least for the common peers served by a particular super peer). As super peer*s* manage subsets of peers in the network, they are more attractive targets for attacks. “The main vulnerability of federated systems are such assumptions that federated service providers (e.g., super-peers) will largely act honestly” [26].

#### P2P PHS Architecture

**Figure 3:**
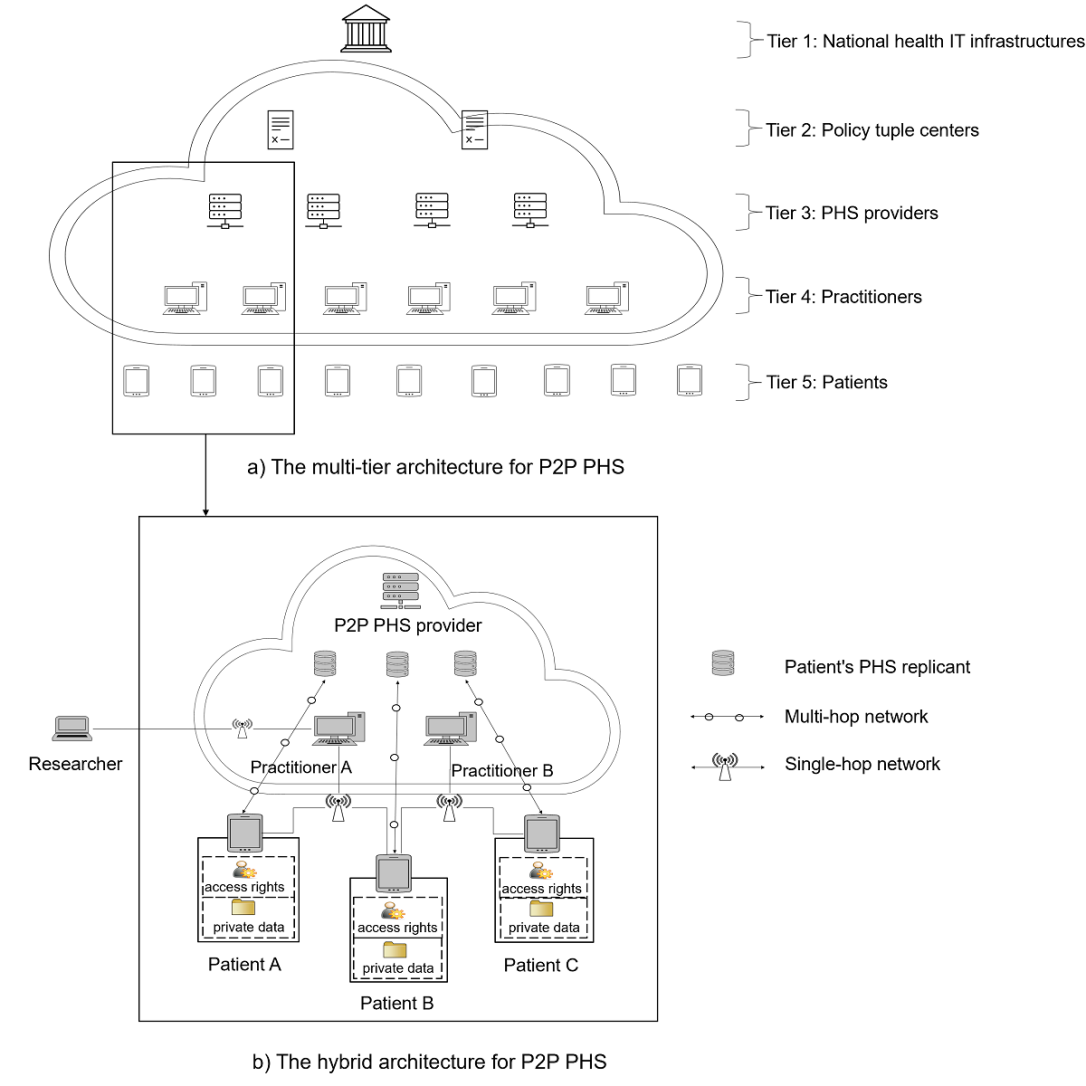
Proposed peer-to-peer (P2P) high-level architecture for patient-centered health care information system (PHS). An aggregate relationship exists between the practitioners and the patients. The patients control the access to their health data, and other entities require patient permission to access a patient’s medical data, for example, by using tokens as currently being implemented in the MedicalChain PHS project [11].

On the basis of the discussion of the different forms of P2P PHS architectures in the previous section, the combination of multitier structure and hybrid P2P architecture appears to be most appropriate for P2P PHSs; therefore, we propose an architecture with the following abilities (Figure 3): (1) enforcement of data protection requirements similar to that of HIPAA and semantic compliance through *super peers* as central index servers; (2) registration and identity verification; (3) higher scalability and availability of resources and lack of single points of failure; (4) association of patients (tier 5, Figure 3) with their respective PHS providers (tier 3, Figure 3) and practitioners (tier 4, Figure 3); and (5) faster PHS updates with security patches through the *super peer* networks. The P2P PHS network is an overlay of the modeled hierarchical relationships between the tuple center and PHS providers, PHS providers and practitioners, and practitioners and patients.

Large health care IT organizations (eg, the German Healthcare Technology Infrastructure; HTI [2,65]) are represented at the top of the hierarchy in the architecture to facilitate certification of various PHS providers (tier 1, Figure 3). They define and enforce the implementation of various data regulations, representation standards, and ontologies (eg, Health Level Seven and Fast Health care Interoperability Resources [6]) to share heterogeneous medical records across PHS networks. In the second tier, a distributed public network of tuple centers (eg, certified through a national health agency) is provided by trusted third parties (tier 2, Figure 3). Agent-based systems (as in centralized P2P PHSs [51]) can be used across P2P networks with the tuple centers' action-reaction rules for communication events [51]. Agent co-ordination models can handle services for data semantics and peer lookup services while serving as mediums for data sharing between P2P PHS providers, but the actual inter-PHS communications are performed in a P2P manner. P2P PHS providers can subscribe to any certified tuple center. Communication of a PHS provider is limited to communication with other subscribers to the PHS provider’s tuple center subscriptions.

PHSs can be provided by any party. In our scenario, we exemplify hospitals (*hyper peers—*managers of super peers and other peers in the network) as PHS providers. The *hyper peers* relay requests and responses among all subpeers across multihop networks. Each *hyper peer* has its own separate private cloud server, which stores a digital and secure copy of patient health records (Figure 3). These records are a replica of the data available on the patient’s local storage but are only made available in the *hyper peer’s* private cloud if a patient subscribed to the corresponding additional PHS features (eg, for data backup, ease of remote data sharing, or emergency access). Accessibility and availability traits of the stored *common peers’* data on the private cloud are in the control of patients through their local PHS client software. This topology can have 2 issues: (1) similar records of patients are stored locally on their mobile devices and the cloud, which appears redundant, but this redundancy curtails connectivity pitfalls while preserving P2P PHS features in terms of offline capability, and (2) the cloud storage can become inaccessible when the local patient PHS device is lost when the device is used as the source of patient identity verification and access authorization for cloud storage.

Each *hyper peer* has multiple health practitioners in the network, which maintain patients’ public identities (under the control of DHT [55,66]) for lookup functionality and ease of data access; therefore, a patient (*common peer*) can be associated with multiple practitioners from various *hyper peers* (practitioner A, B, C, etc). In such cases, these *hyper peers* can communicate via tuple centers. This way patient data stored on a cloud of hospital B can be accessed by practitioners in hospitals A or C for diagnosis or treatment, given that the patient grants access rights. Each *common peer* on the network (corresponding to a patient) is modeled on the local PHS and on the *hyper peer’s* private cloud server. *Common peer*s can grant access to their health records to any party through single-hop radio communication (without involving a third party in the communication, eg, Wi-Fi direct) or multihop network communications via the cloud storage of the *hyper peers* [65]. Other parties, such as researchers looking for data for research purposes, can obtain read-permissions for patient records by interacting with the practitioner via the hospitals' private network, which forwards permission requests to patients. However, only aggregated results (anonymized) are returned to the researcher. Moreover, wearable mobile devices and biotechnologies that provide biometric or psychometric data can also be directly connected to a patient’s P2P PHS.

## Methods

### Literature Search

**Figure 4:**
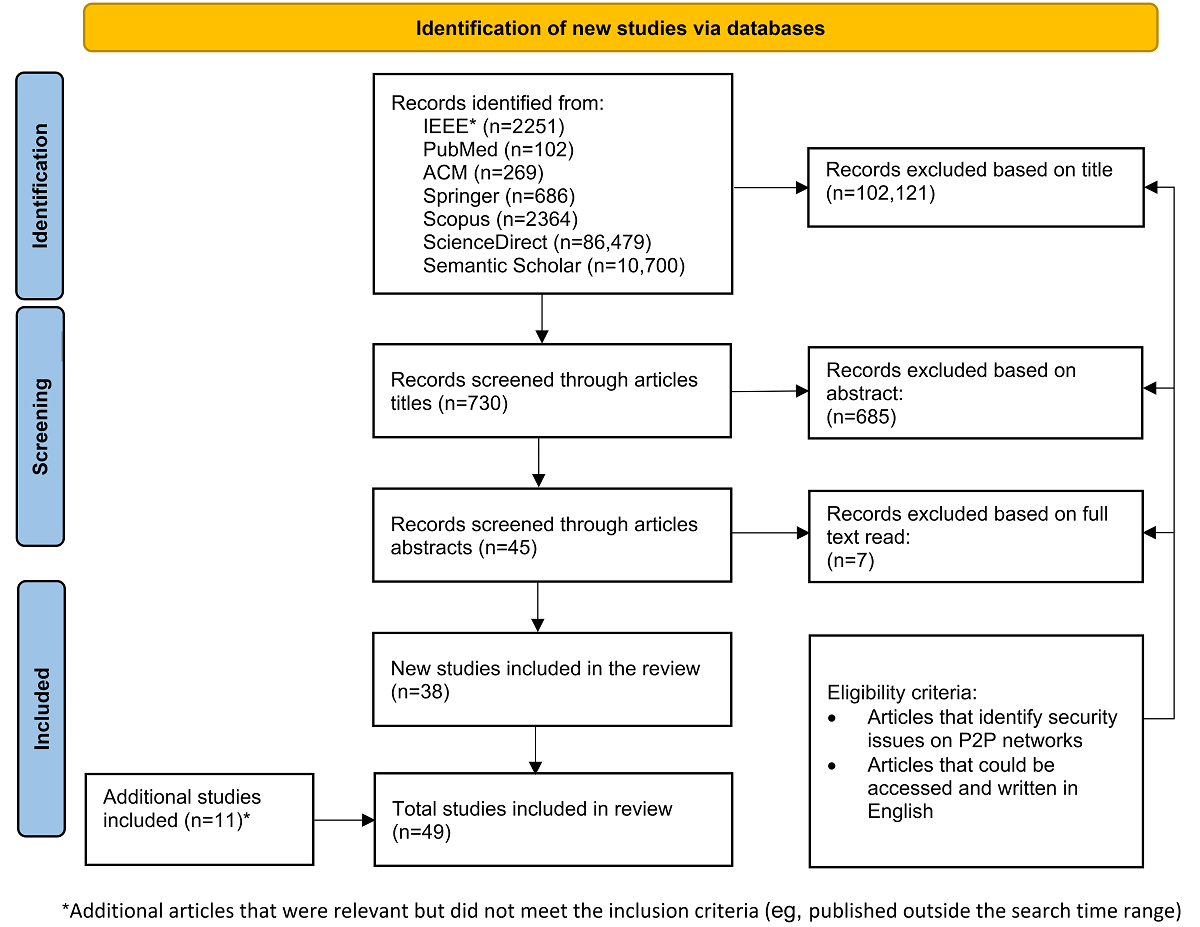
PRISMA (Preferred Reporting Items for Systematic Reviews and Meta-Analyses) flow diagram. P2P: peer-to-peer.

We conducted a systematic literature review (Figure 4) following the PRISMA (Preferred Reporting Items for Systematic Reviews and Meta-Analyses) reporting guidelines [67,68] and used thematic analysis to guide the data analysis process [69]. The systematic literature search in this study was conducted using specialized academic search engines (IEEE Digital Library, PubMed, Science Direct, ACM Digital Library, Scopus, and Semantic Scholar; see Multimedia Appendix 1 for further details). The search was conducted on articles published between 2008 and 2020. The study selection was organized into the following phases.

1. The search string was derived by breaking down the research question into different facets, where their alternative definitions and acronyms are included and combined using the logical operators “OR” or “AND” [68]. The search string “(*P2P* OR *Peer-to-Peer*) AND (*vulnerabilities* OR *vulnerability* OR *threats* OR *threat*)” was applied to the title and abstract and adapted to the specific syntax of the used search engines.
2. Eligibility criteria: we included all articles that could be accessed, were written in English, were published in academic outlets, and identified inherent security issues for PHS deployment on P2P networks, as suggested for thematic analysis [69].
3. Abstracts of the filtered articles were further analyzed by the authors to remove irrelevant articles based on eligibility criteria and other false-positive results.
4. Articles were grouped and duplicates were removed.
5. The remaining articles were read in full text and analyzed by the authors (assisted by Atlas.ti software [70] to manage codes and themes for thematic analysis [69]) to include only relevant studies based on the eligibility criteria defined in step 2.

### Identified Articles

Initially, 102,851 articles were identified using the search string. The filtered articles were screened based on their titles using the same search strings. A total of 99.29% (102,121/102,851) false-positive results were removed. Further examination of the abstracts of the remaining 0.71% (730/102,121) articles resulted in the exclusion of 0.67% (685/102,121) articles. The main reason for exclusion in this step was a lack of thematic fit with our study (eg, a focus on P2P currency exchange or lending platforms or security issues for largely unrelated technologies such as robotics). We analyzed the full text of the remaining 0.04% (45/102,121) articles, and 0.01% (7/102,121) further articles were excluded. We complemented the result set with 0.01% (11/102,121) additional articles that met the eligibility criteria but not the inclusion criteria (eg, published before 2008). Ultimately, 0.05% (49/102,121) articles remained.

### Thematic Analysis

Data analysis was guided by thematic analysis [69] to identify the relevant themes in the identified articles. The initial coding was performed by the first author and refined and finalized in group discussions with the other authors. The themes (codes) were identified using the key security goals (theory-driven) from the CIA (ie, confidentiality, integrity, and availability) triad as organizing codes for data analysis (assisted by Atlas.ti software [70] to manage codes and themes for the thematic analysis). *Confidentiality* entails that unauthorized actors cannot access information during transmission, processing, or in storage. *Integrity* requires that the information not be modified unintentionally or without authorization. *Availability* means that the system is accessible to the user when needed. For each of the codes identified, we looked at the impact of the security issues associated with the codes to examine their impact on P2P PHS (eg, potential for unauthorized access). We then investigated and rated the consequences of potential exploits of P2P-PHS security issues based on the Common Vulnerability Scoring System (CVSS; see Multimedia Appendix 2 for further details).

The systematic literature review revealed 8 main P2P security issues (list of themes) extracted through data analysis and 7 factors promoting them. Table 2 shows the summary—generated codebook—of the security themes identified along with their sources and exemplary codes used to derive the themes during the thematic analysis process.

**Table 2:** Overview of peer-to-peer security themes identified^a^.

Combined themes, second-order themes, and first-order themes				Study
**Pollution**				[71-81]
	Metadata pollution	Changing original file name or extension Replacing the file with a misleading one		
	Index pollution	Claims ownership of wanted but bogus content Sharing of the content record via the index		
	Content pollution	Modifying the file content Replacing the file with an incorrect one		
**Malware**				[78,81-91]
	Virus	Infection of the system Appears to be part of legitimate programs		
	Spyware or ransomware	Spying or stealing user data Encrypts any kinds of files and data		
	Worm	Infection of P2P^b^ routing table Appears independent of existing programs		
**Social engineering**				[35,80,82,92-95]
	Baiting	Tricks user to divulge sensitive information Relies on human error or mistakes		
	Phishing	Scam via email or SMS text messages Trick into divulging sensitive information		
**Poisoning the network**				[35,43,45,47,56,71,73,77,81,89,95-102]
	Index poisoning	Sharing of bogus contents via indexing table Affects network quality of service		
	Routing table poisoning	Sharing of bogus contents via routing table Prevents from finding correct resources		
**Sybil**				[26,52,54,56,63,72,76,81,84,92,103-112]
	Faking identity	Faking multiple identities for a single user Affects the redundancy property of P2P systems		
	51% attack	Outvoting of honest nodes in the network Cheating without being detected		
**Eclipse**				[47,54,56,72,77,79,81,92,93,105-108,113-116]
	Large man-in-the-middle	Separating the network into several portions Acts as gateway and disrupts message flow		
**DDoS^c^**				[43,45,72,76,77,80,81,84,88,92,94,95,97,98,100,102,105,110,117-119]
	Flooding	Invalid packets flood the network Impedes delivery of normal packets		
	TCP-DDoS^d^	Connection overload with full TCP-requests Denies connections from legitimate requests		
**P2P traffic blockade**				[46,100,120-122]
	Port number blockade	Blocking of P2P network traffic Imposes bandwidth limits with P2P networks		

^a^The first- and second-order themes are only examples and not exhaustively listed.

^b^P2P: peer-to-peer.

^c^DDoS: distributed denial-of-service.

^d^TCP-DDoS: transmission control protocol–distributed denial-of-service.

## Results

### Factors Promoting Security Issues in P2P Networks

To use a P2P network for resource-sharing, multimedia-streaming, distributed-computing, or telephony applications, users install a P2P application on their device and permit the application to access and use device resources such as cameras, microphones, or device storage. In P2P operation, the P2P client application reads files from the user’s disc during the uploads and writes to the user’s disc during download. During this operation, personal or sensitive information can be transmitted to the network.

#### Inadvertent Sensitive Information Disclosure

It is often not necessary that users’ confidential or personal documents be exposed by worms or viruses, as many users inadvertently expose these documents [123]. For example, a node may request data X from the user, and the user sends back the entire folder where data X is located. The user may end up exposing all of their sensitive information for the following reasons: (1) a user does not appropriately select or share the requested data, (2) the interface design of the P2P application confuses the user, and (3) the requester offers a huge incentive to share. In 2012, an automated personal health information tool was used to crawl different P2P networks (FastTrack, Gnutella, and eD2K) to analyze Canadians’ personal health information and personally identifiable information in the exchanged text files [83]. Out of the 3924 P2P files with unknown content, 1.45% (57/3924) of files were flagged as personally identifiable information. Manual analysis of the 57 files revealed that 19% (11/57) contained health information about an identifiable individual, that is, inadvertently disclosed health information.

In 2019, a survey identified human errors, such as sending personal information to unintended email recipients or releasing personal information by accident, as the largest source of data breaches in the health sector [39]. Similarly, several peers were found to be inadvertently sharing their financial, email, and web cache data in a study on the KaZaA P2P network [124]. In addition, some P2P users share their personal information intentionally to increase the number of files shared on the network to meet the participation requirements of some P2P systems [85].

#### Set-and-Forget

P2P clients tend to be set-and-forget applications that run in the background [85,123,125]. This means that the user is not cautiously tracking the activities of the P2P client, which increases the opportunity for abuse.

#### No Borders

**Figure 5:**
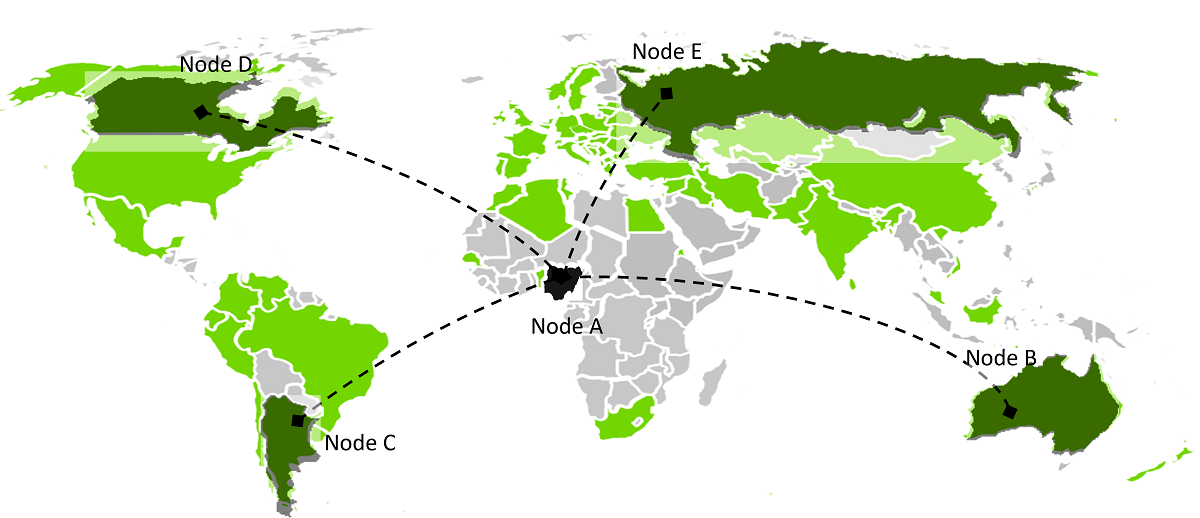
Geography example of a peer-to-peer (P2P) network.

Geography is largely irrelevant in P2P networks [85], and no region is safer than the other. A computer in Australia or Argentina becomes part of the same network as a computer in Nigeria (Figure 5). In open P2P networks, files can undoubtedly migrate globally, and threats can come from any region of the globe. Hence, the heterogeneity and geographically dispersed nature of P2P networks can be a problematic factor affecting security, quality of service guarantees, and scalability. However, studies have shown that P2P networks converge to a certain degree of geographical clustering [85,126]. Users may choose to download and share content from their region to have lower network use and latency than when downloading or sharing content outside their region.

#### Growing Use and Network Heterogeneity

As a P2P network grows, an increasing number of leaks of confidential files will occur in the network. In 2017, nearly 27 million P2P users downloaded and shared files on P2P networks daily, which is 17 million more users than in 2006 [127,128]. Moreover, P2P networks are heterogeneous and fast-moving; hence, users may not be able to keep track of security issues and developers may neglect them [85].

#### No Content Verification

Conventional P2P networks have no trust mechanism to assist users in deciding whether to share or download content in the network. Similarly, they have no central authority responsible for verifying the authenticity of the resources shared by users [80]. Hence, there is no guarantee that users are sharing the content they promise. This makes it easier for an attacker to spread malware across a P2P network, for instance, to conduct fraudulent activities or pollution attacks [72].

#### Digital Winds Spreading Files

Typically, P2P networks create file indexes using the names of the files and the associated metadata [123]. This constitutes a security issue, as it allows anybody to easily discover files in P2P networks. For example, an opportunistic search with key terms related to the top 10 publicly traded health care firms in the United States revealed 20,000 patient records, 4 patients with acquired immune deficiency syndrome (AIDS), 201 patients with a mental diagnosis, and 326 patients with cancer [125]. The approaches that some P2P clients use to create and manage file names have serious implications in exposing users’ private and confidential information. This can be a problematic factor regarding security because users’ sensitive files can be easily discovered owing to poor P2P client design.

#### Snooping Nodes

This factor enables attackers to leverage the open nature of P2P networks [100]. The long routing paths across several nodes create a loophole for malicious activity [94]. Peers in a privileged position in the network (eg, *super peers*) are able to see the communication of other *common peers* in the network. For example, decentralized P2P systems such as Gnutella [35] have no central servers or auxiliary mechanisms to co-ordinate communication among users, but when a new user connects to the Gnutella network, it chooses a node as its permanent entry point [115]. Thus, high-speed nodes are inadvertently placed in the central part of the topology and can observe the communication of nodes in their local subgraph. Moreover, communication in P2P networks stops being anonymous as soon as the source node establishes a direct connection to a destination node to download files [35]. The IP addresses of both nodes are exposed to each other, which creates another opportunity for abuse. Once the identity of the peer is revealed, further attacks can be carried out [96].

### Identified Security Issues and Their Impact on P2P PHSs

#### Pollution

Pollution is a form of attack in which an attacker modifies the original content (through mixing or substituting) so that it has no use or is of low quality [72,79,81]. The polluted content appears to be legitimate content (eg, by having a similar size, format, and title) to trick users to download it. However, the altered content may be malicious, fake, or corrupt. This affects the network’s quality of service (especially in file, voice, and video-based P2P streaming systems [72,73,75,79,80]), overall system energy consumption [74], content availability [78], and data integrity [72]. Pollution is an easy and fast way to disseminate worms or viruses from one to many peers in the network. Therefore, pollution can have an exponential impact on the security of the entire network [72]. The pollution attack was first discovered in 2005, where a crawler was used to retrieve super peers in the KaZaA P2P network [73]. Analysis of the contents collected by the crawler revealed that over 50% of welcome copies (ie, introductory files for a collection of files) for musical files in the KaZaA network were polluted [73]. Pollution is a serious attack on P2P networks, even in a scenario with only one polluter [72,75]. The impact grows when the number of polluters or peers attempting a request increases [75]. As a result, peers often require multiple times the network bandwidth they need in a network free from pollution [75]. Furthermore, the attack is persistent. Even if the polluted contents are identified and blocked by the network, the polluters may remain alive in the network by disguising their identities and can keep polluting the network.

Pollution is categorized based on the attackers’ strategy: (1) metadata pollution, where a file extension or name is modified and replaced with a misleading one; (2) content pollution, where the file content is changed; and (3) index pollution, where an attacker claims ownership of an unindexed bogus file and uploads its record (IP address, port number, etc) to the entities (eg, super peers on hybrid P2P) that maintain such records for distribution [73,77]. In most cases, the polluters also attack legitimate peers’ reputations or boost their own reputation through whitewashing attacks [75,76]. Content pollution is the most popular and common attack in P2P streaming systems [74]; it was detected in 50%-80% of files in KaZaA and about 50% of popular files in eDonkey [73,74]. Pollution is not necessarily caused by malicious users; P2P systems are notorious for illegally sharing and disseminating copyrighted content, and content is often polluted by copyright owners as a countermeasure to protect their rights when legal actions fail [71,72]. To facilitate the protection of copyright claims, some P2P system providers even weaken protection from pollution attacks in their network [73], although this affects the confidence of users in such systems [72,73].

#### Impact of Pollution Attacks on P2P PHSs

Successful pollution attacks on P2P PHSs can be devastating because of the higher integrity and availability requirements of medical data than data shared in other P2P systems. The consequences of its exploitation could be between low and high, depending on the level of access gained; pollution attacks often serve as a gateway to identify vulnerabilities (eg, unverified inputs that can be used for SQL injection attacks [129]) and mount further attacks (eg, ransomware attacks). For example, in 2020, a patient in need of emergency care due to an aneurysm died in Germany during a ransomware attack in a hospital. The ransomware attack caused a network outage that disrupted emergency services, and the patient was sent to a health care facility approximately 20 miles away [130]. This diversion delayed the treatment of the patient by an hour and she died [130]. The openness of P2P systems allows polluters to easily join and leave the network [20,56]; however, identity verification (eg, via insurance, job contract, token, etc) and multifactor authentication concepts for P2P PHSs could create an additional layer to reduce the vulnerability of the network. Patients or practitioners polluting a P2P PHS through their legitimate accounts can easily be traced; however, in some situations, a double-faced user (legitimate but malicious) could leverage open-source hacking tools such as Burp Suite [78] to, for instance, alter an http request payload with an anonymous ID, add polluted content, and forward it to the content distribution network of a hospital to harm the network.

#### Malware

Malware refers to a wide range of attacks that compromise a system without the knowledge of the system owner [84,90]. P2P networks present a greater risk for receiving malware; for example, only 3 strains of malware infected over 68% of compressed and archived files on the Gnutella network [84]. In the first 3 quarters of 2019, 7.2 billion malware attacks were reported globally [91]. In P2P networks, malware is predominantly used to create botnets by leveraging worms [84,89,90].

A botnet is a network of infected nodes that are usually compromised by worms or viruses. Individual bots in the botnet only use a small portion of the infected resource to remain concealed and create only barely noticeable traffic to share data from the compromised computers with the target [88,89]. The bots are controlled by an attacker (botmaster) through command-and-control servers [89].

A worm is independent and neither requires a host application [84,87,92] nor human intervention [82] to propagate and replicate itself over a network. Worms can result in a high fallout in combination with other vulnerabilities and propagate themselves over email attachments, web server infections, file downloads (counterfeit worms), or other legitimate network activities (silent worms) [78,81,82,84,87]. Passive (counterfeit and silent worms) and active worms are 2 broader categories of P2P worms; they both propagate like a biological virus, but the former waits for victims to infect, while the latter actively searches for new targets [84]. The threats to the amplification of worm-based attacks in a P2P network are high, and the impact grows based on network size, topology degree, or host vulnerability [78]. In contrast to the internet, where worms need to randomly search to identify vulnerable hosts, P2P worms spread rapidly and infect all nodes in the network almost instantaneously [84]. For example, the Antinny (passive and counterfeit) worm that appeared on the Japan-based Winny P2P network led to the disclosure of a large amount of private data: thousands of patient health records, customers’ identifiable information, top-secret military information, and documents of a county police investigator, yielding information on major investigations on 1500 individuals [85,86]. Furthermore, in 2001, in less than 14 hours, the Code-Red worm (active) infected over 350,000 systems and caused more than US $1.2 billion in damages in the first 10 days of its circulation [78].

P2P worms are some of the best facilitators of botnet-based attacks and internet worms. P2P networks are, for instance, known for sharing *gray* content, such as pornography and pirated streaming media. This can lead users to incautiously monitor unusual behaviors in the network [78,84,85]. Active P2P worms have different attack strategies: pure random scan (PRS), offline hit-list scan, and web-based scan [78,82,84]. The PRS is a starting point, information gathering stage, and is the most commonly used strategy [78]. PRS is useful when the infected host (bot) possesses no prior vulnerability information of potential targets and randomly selects and mounts attacks on targets to propagate the infection, for instance, using random IP addresses searched from the global internet address space [78,82,84]. The offline hit-list scan is a more powerful strategy: the attacker collects and continuously attacks targets using DNS, network topology, and routing information of P2P systems (eg, using crawler tools [83]) until all the hosts in the hit-list are scanned, and the newly compromised bots attack using the PRS strategy [78,82]. Instead of an offline hit-list, the web-based scan strategy primarily launches attacks on its web-based P2P neighbors, and then the worm disseminates further using PRS through the infected worm hosts [78,82].

#### Impact of Malware on P2P PHSs

Ransomware constitutes the biggest threat with 151.9 million attacks globally in the first 3 quarters of 2019 [91]. Moreover, ransomware attackers are shifting tactics to target higher-value institutions, such as hospitals [91]. In 2017, a malware was used in the WannaCry ransomware attack, which infected more than 230,000 computers worldwide [131]. In the British National Health Service, WannaCry disrupted scheduled treatments in many hospitals, resulting in total damages of around £92 (US $12.6) million in the United Kingdom [132]. The malware hijacked users’ data, encrypted the data, and blackmailed users before decrypting their data [133]. For health data on P2P networks, which have a less controlled infrastructure, ransomware attacks can become easier.

The effect of malware on P2P PHS could be high, although the severity of malware attacks is context-dependent. The effect of malware, such as Antinny [85,86], Anatova [134], or Code-Red [78], on P2P PHSs will be detrimental if it denies patients and physicians access to the PHS, steals patient data, or hijacks and encrypts data for ransom. Structured P2P PHSs, similar to our proposed architecture (Figure 3) or the e-toile framework in Switzerland [21], could be less vulnerable to malware in comparison with unstructured P2P PHSs. This is due to the possibility of using control measures on the index and DHT networks [55,66]. The factors that increase the attack surface include that P2P client applications tend to be *set and forget* [85,123,125] so that they run in the background while the user is not monitoring its activities and that there is no centralized control to detect and prevent attacks in P2P networks. The impact of malware could also escalate beyond the boundary of the P2P network and impede usability features such as emergency access or guardian support. In P2P PHSs, these disruptions can occur on a greater scale than in the example in the previous section, where a single patient could not be treated in a hospital because of a ransomware attack [130].

#### Social Engineering Attack

Some P2P clients are being used by users with limited knowledge of computers and information security [80,94,95]. Depending on the nature of the target network, the effect of social engineering attacks—an attack on the users involved in a system [93]—can facilitate exploits of other vulnerabilities. P2P worms such as silent worms (eg, VBS.Gnutella worms [82]) are based on social engineering, disguise themselves, attach to a known file, and wait to compromise victims [93]. Moreover, some P2P systems (eg, Napster and BitTorrent [92]) implement mechanisms in which the users are incentivized to share resources or content to gain greater performance and access to content; therefore, experienced users or attackers can exploit the eagerness and likely incautiousness of new users to deceive them and obtain confidential information, which could be used to conduct malicious attacks. Owing to the *set-and-forget* nature of P2P file-sharing applications [35], users may not realize the breach of confidentiality risks when using them, which increases the chances of abuse.

#### Impact of Social Engineering on P2P PHSs

Social engineering can affect all types of P2P PHSs, where an attacker can easily leverage the user layer to deceive patients (older adult patients are more vulnerable to this attack than others [135]). In the case of P2P PHSs, the threat impact could be one user at a time, with the probability of escalating and affecting others in the network. Social engineering can be observed as an intelligent information gathering stage for attackers to mount other attacks [129], such as scamming patients to obtain, for instance, access credentials to their P2P PHS accounts. Depending on the attackers’ goals, they may modify patients’ health records or upload malware to the P2P network to affect patients’ lives, health, location, privacy, behaviors, or activities [93] and sabotage the PHS and its providers.

#### Poisoning the Network

Poisoning can be performed either by *index poisoning* or *by routing table poisoning* [102]. Many P2P systems have a lookup service using indexing or routing table techniques [35,47,95]. A poison attacker can use this to inject invalid information such as bogus resource identifiers or fake IP addresses into the lookup service. An index poisoning attack affects the index of P2P systems [43]. Injecting invalid information in the index or routing table can slow down the query, prevent others from finding the correct resources, or result in a peer wasting time connecting to invalid peers [100,102], which eventually affects the P2P network’s quality of service [101]. Some anticopyright infringement organizations use poisoning attacks to prevent the sharing of pirated content on P2P networks [89,99,100]. These attacks are performed by identifying and poisoning the IP addresses of the servers for pirated content or using their IP addresses as evidence to sue the content server or P2P system providers [71].

An index maintains records in a centralized manner (eg, Napster [50], P2P PHR [6], or e-toile framework [21]) and enables users to locate resource owners’ IP addresses and port numbers. In *index poisoning attacks*, the attacker aims to compromise indexing peers (peers that participate in the indexing) by adding invalid information into their local indexes by simply sharing the bogus information with the indexing peer [43,81].

A poison attacker can also attack a specific host; for example, if the attacker wants to conduct a DDoS attack on the application server at host 129.13.152.6, the invalid information may include 129.13.152.6 for the IP address and 80 for the port number. Once the indexing peer has been poisoned, another peer can search for a resource and eventually receive invalid information from the poisoned peer and try to download the resource from the victim host. Before downloading the resource, the transmission control protocol (TCP) connection is established with the victim host using invalid information. To download the resource, the requesting peer sends a message to the desired resource. When many peers try to download the resource from the victim host, a TCP-connection DDoS comes into effect [43,97,98].

Structured P2P systems (eg, P2P IHE [51], our proposed PHS architecture [Figure 3], Chord, and Kademlia [35]) are vulnerable to poison attacks [95], although resource discovery is under the control of data structures (eg, DHT). In *routing table poisoning*, the poison attacker exploits the fact that each peer in a DHT-based P2P system maintains the routing tables of its neighbors [47,56,73,77,95,96]. Each entry in the table includes the neighbor’s identifier, IP address, and port number. The attacker can deceive participating peers by injecting invalid neighbors into their routing tables. The poisoned peer may choose an invalid neighbor in its routing table and forward its messages. If the routing tables of many peers are poisoned with invalid information and each entry points to the IP address of the victim host, the target receives a flood of messages from the DHT [95]. A further type of content pollution attack is a *combination attack* that combines *index poisoning* and *fake-block* attacks to have a higher impact [45,77]. In this case, poison attackers use an index poisoning attack to include their IDs in the invalid information to be advertised. If the victims establish the connection through the invalid information, they may connect to a poison attacker, so that the attacker can feed the victims with fake fragments and impose more harm on them.

#### Impact of Poisoning Attacks on P2P PHSs

Centralized P2P PHSs, such as P2P PHR [6] and the e-toile framework [21], could suffer the worst effects of poison attacks because they can cause DDoS or entire network failure and disrupt the services offered by PHSs. For example, in the e-toile framework [21], a list of health care stakeholders and their access rights, data exchange, and authentication is managed by a central index server; poisoning such an index could mean that the data of a patient registered with PHS^X^ in need of emergency care at a remote hospital that uses PHS^Y^ could be inaccessible to practitioners. Even if the networks of PHS^X^ and PHS^Y^ are not affected, the single point connecting the PHS providers is disrupted. Depending on the urgency of a patient’s need for treatment, the need for access to health data, and the longevity of the attack, the patient’s health and life could be adversely affected. In some P2P PHSs (eg, P2P PHR [6] or P2HR [20]), peers’ IP addresses are exposed to facilitate health information exchange between different health entities; this makes the attack even easier. For our proposed P2P PHS architecture (Figure 3), there is a federation of PHSs and tuple center providers. Within the context of the previous scenario, access and data exchange will not be impacted if PHS^Y^ is in the same tuple group as PHS^X^.

#### Sybil Attack

The name Sybil attack was coined by Microsoft Research in 2002 based on the book Sybil about a patient, named Sybil, diagnosed with dissociative identity disorder [111]. In computer security, Sybils refer to multiple identities of a single user on the same machine; this user can become powerful and control a significant part of the network or use the identities to influence the system behavior [54,56,81,109,110,112]. In DHT-based P2P systems, a user can locally generate multiple *node IDs* for many node instances on the same machine [108]—on the Kad network, a single node can select multiple IDs concurrently [107]. The creation of Sybils is considered the most harmful behavior on a P2P system [54], as it offsets the network’s redundancy property [81]. Sybil attacks occur in a P2P network, when the reputation mechanisms are compromised [72], secure authentication mechanisms are not implemented (eg, no proof of identification is required for registration in the P2P session initiation protocol network [106]), or verification of a client’s IP address and its maximum number of connections per ID is not implemented (eg, Kad network [98]). Limiting the number of connections per IP address (eg, in eDonkey [84]) does not prevent Sybil attacks because attackers can bypass this by having many virtual IP addresses. It seems that there is no clear and definite solution to prevent Sybil attacks [26]; this is due to the openness and lack of admission control mechanisms in P2P networks.

**Figure 6:**
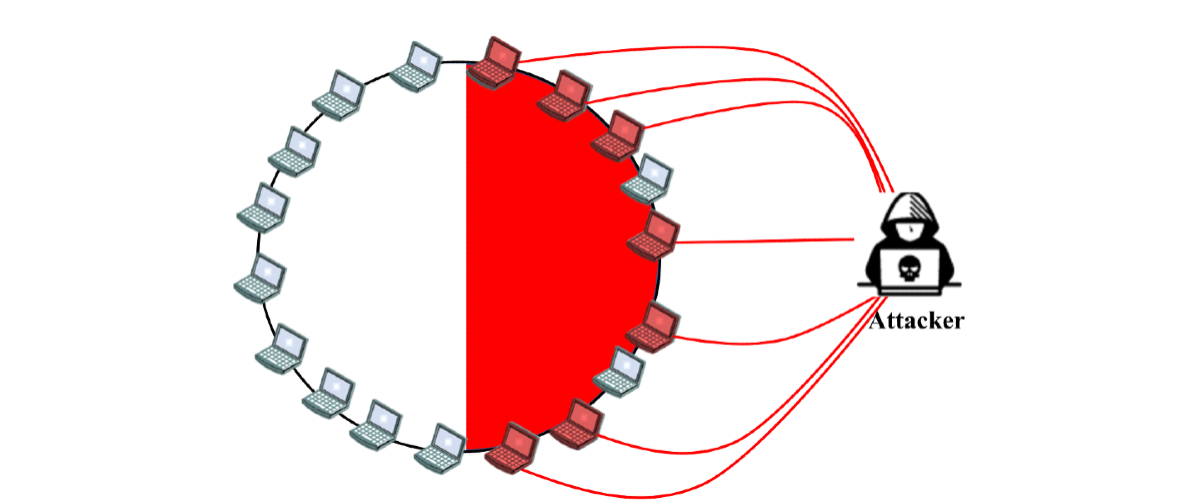
Example of Sybil attack [92]. The attacker placed his malicious nodes on one side of the network segment. Placing many malicious nodes in the network enables the attacker to gain control of the activities of one-half of the network.

Sybils are used by attackers to conduct massive and organized attacks on P2P networks [92]. For example, eclipse attacks [54] amplify Sybil attacks through the combination of Sybil and ID assignment or mapping attacks [105], which assigns identifiers near the same portion of the ID space to sufficient Sybil nodes (Figure 6). This enables the attacker to own a deciding power of where in the ID space the new nodes are placed. When the attacker owns more nodes than the benign nodes in the segment, the attacker can control messages in the segment, bias reputation score, create DDoS situations, or force servers to exceed their CPU capacity [26,76,84], which is also known as a gateway attack [92]. In blockchain P2P networks, Sybil attacks are, for instance, used by attackers to outvote the honest nodes in the network [52,63,104], which enables the attacker to cheat without being detected. After a successful Sybil attack, attackers can transmit or discard blocks, effectively block other users from the network, carry out *51% of attacks* to change the order of transactions, prevent transactions from being confirmed, or even reverse transactions that they made, which can lead to double spending [103].

#### Impact of Sybil Attacks on P2P PHSs

Sybil attacks are helpful for attackers to disguise their identities, access vital information managed in the PHS index service, monitor communications between users, steal patient data, or pollute the entire network to disrupt the entire PHS service operation, which would affect patients’ health and life and sabotage the PHS provider's reputation. In our proposed PHS architecture (Figure 3) or the e-toile framework in Switzerland [21], the national health IT agencies are tasked with effectively handling health care stakeholders’ registration, authentication, and verification; therefore, freedom to create multiple concurrent IDs on the same system by any malicious user is reduced by design. P2P PHSs, such as P2P IHE [6,51], could be more vulnerable to Sybil attacks due to the difficulty in establishing control mechanisms in a decentralized network. In any case, attackers can leverage Sybil attacks to steal patients’ identities (eg, for insurance coverage or blackmail).

#### Eclipse Attack

**Figure 7:**
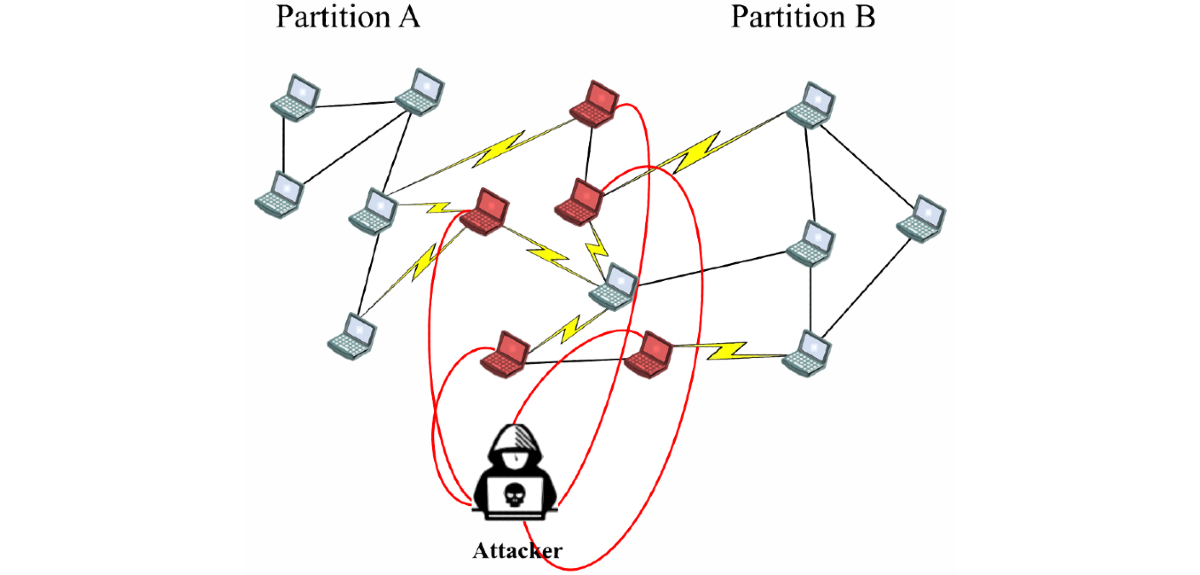
Example of an eclipse attack [92]. The attacker successfully segmented the network into 2 ID spaces. The communications between the nodes in the network must be forwarded by the malicious nodes.

An eclipse attack is a large-scale man-in-the-middle (MitM) attack that is commonly executed at the P2P network level [54,92]; routing, sniffing, and traffic analysis attacks are variants [56,79,81,93,105,106,115,116]. An eclipse attack aims to separate the entire network into 2 or more partitions (Figure 7) by placing malicious nodes in a strategic routing path of the P2P network [105,106,108] to surround benign nodes with malicious neighbors [77]. In most cases, the routing mechanisms are attacked [47]. This is accomplished by adding the attackers’ addresses to the neighbor list of the benign nodes [54,81] or through fake routing updates and incorrect routing [105]. Once the network is fully segmented with malicious nodes in between the partitions, the attacker can act as a gateway and disrupt the information flow between the network partitions, exclude groups of nodes from the network, or steal peer identities [54,77]. This affects the reliability, autonomy, and connectivity between peers and the CIA properties of P2P networks [72,106,114]. In addition to mounting an eclipse attack by manipulating the overlay network, an attacker that has collected a significant number of peer IDs and acts as a neighbor of benign nodes can easily mount eclipse attacks [54,77,81,107].

Successful eclipse attacks require attackers to possess a high proportion of fake nodes in the network and a higher number of direct routes coming to their nodes than to the average benign nodes in the network [54,77,81], especially in networks with relaxed rules for maintaining the routing table [92]. P2P systems that have no control over node placement in the ID space (eg, Gnutella [54]) or freedom of choice for identifiers (eg, Kad [107]) are highly vulnerable to eclipse attacks. P2P networks are more susceptible to eclipse attacks when they are new [54].

As seen in the Bitcoin network, a botmaster with as few as 24 IP address blocks can eclipse any node with a minimum probability of 85%, irrespective of the number of nodes in the network [114]. Despite new security patches that address eclipse attacks on the Bitcoin network, a novel form of eclipse attack, EREBUS, was found [113], which partitions the network and affects Bitcoin nodes' peering decisions. This shows the likelihood of exploiting eclipses in P2P networks.

#### Impact of Eclipse Attacks on P2P PHSs

The lack of freedom to select and place identities and the presence of a control infrastructure in centralized and hybrid P2P PHS (eg, our proposed architecture [Figure 3] or the e-toile framework in Switzerland [21]) reduces the impact of any form of eclipse attack on P2P PHSs. This could be higher for decentralized P2P PHSs such as P2P IHE [6,51] because of the absence of centralized trust and control infrastructures and the presence of eclipse attack vectors such as resource routing mechanisms in the network [47]. In addition, a successful attack could allow an attacker to eavesdrop on the conversation between users in the network without potentially compromising the patient's system. P2P PHSs on a patient device can be configured with wearable smart sensors to allow health practitioners or an embedded machine learning model to monitor vital parameters (eg, heart rate variability). In the case of a successful MitM attack on such P2P PHSs, the practitioners or machine learning models may receive unreliable data, which could lead to poor therapeutic or diagnostic decisions and even loss of life [93,135]. An attacker can also share fake messages that an older adult has fallen in order to summon the next-of-kin or emergency services or use the patient's location or personal data for blackmail [93,135].

#### DDoS Attack

**Figure 8:**
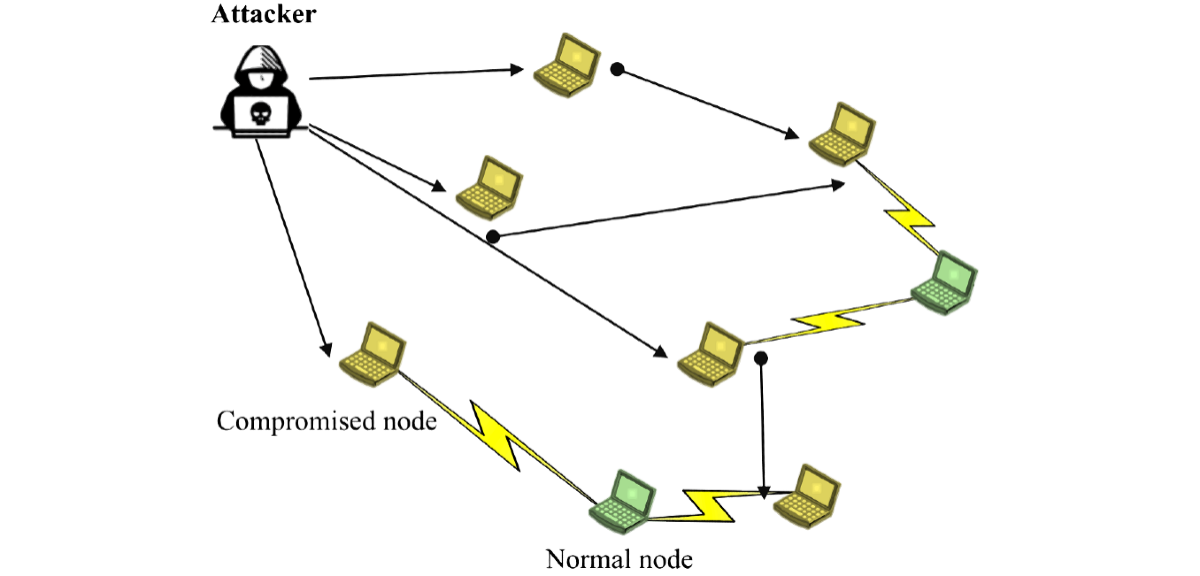
Example of a distributed denial-of-service (DDoS) attack [92]. The attacker successfully executed the DDoS attack and compromised many nodes in the network. The normal nodes cannot establish connections to other normal nodes.

A traditional denial-of-service (DoS) attack stops a service [92,94]. Query flooding is the most common resource and key to mounting DoS on P2P networks [77,105,117]. Invalid or corrupted packets flood the network [95] and impede the delivery of valid requests or messages in the network—byzantine attacks [119]—and therefore stop all communications passing through the affected routes. A DDoS is said to occur when constant streams of invalid packets flood the network in such a way that a single node has to deal with massive traffic and runs out of bandwidth [43,80,81,92]—bandwidth attacks (Figure 8). A lack of central authority can be the root cause for DDoS [97], but the root cause can also be due to the absence of mechanisms that verify response messages from other nodes (eg, in Kad [98]). Many nodes (or zombies controlled by attackers, where each zombie may control other attacking zombies) participate in DDoS attacks [81,88], while the source of the attack is hidden behind a separate layer or through spoofed IP addresses [84,92,105]. This disguise of the attackers makes it difficult to detect them because they are often only indirectly involved [81].

The previously discussed index and DHT routing table poisoning attacks and file request redirection (or topology change) attacks are other methods of mounting DDoS [77,84,98,102,110,118]. A file request redirection attacker (chatty peer) advertises the possession of many false resources that are rare in the P2P network and then establishes several TCP connections with the victims (requesting peers) [45,100,102]. However, if the requesting peers ask for the blocks of the requesting resource, the attacker only resends handshake messages to the victims and never uploads any blocks. This makes the requesting peers spend much time waiting in vain for the attacker's response and blocking other legitimate users from making connections to them. As such, TCP-connection DDoS comes into effect and affects the availability of entire P2P networks [72]. A request-redirection DDoS attack on internet equipment was used to shut down tech giants’ websites (eg, Yahoo and Amazon) in February 2000 [84], which shows the impact severity of DDoS on any network.

DDoS is an active attack that makes it more aggressive. An attacker often attacks the network to prevent certain users from performing their tasks or put the system out of service in one or many segments of the underlying infrastructure [76,84]. The probability of a DDoS attack is high in large P2P networks because nodes have to be reachable (usually outside of firewalls restrictions, etc) by the network [92,117]. Depending on the number of zombies, DDoS on decentralized P2P networks may barely affect the entire network, except for a certain number of affected peers. On the contrary, the impact could be higher on centralized and hybrid systems because communication relies on a single entity that is reachable throughout the network or subnetwork. The higher the number and diversity of nodes involved in the DDoS, the more difficult it is to be blocked [81,97].

#### Impact of DDoS on P2P PHSs

When P2P PHS providers are hospitals, as in our proposed architecture (Figure 3), and store all patients’ medical records, a successful DDoS attack on the network (index or super peers) will have severe consequences. The effect could disrupt the network and data access and cause a delay in treatment and even loss of life (eg, the case of a patient who died after a malware hit a hospital in Germany [130]). In some centralized and hybrid COVID-19 contact tracing systems (eg, PEPP-PT [22] and Trace-Together [23]), the identifiers (ephemeral IDs) that are used to share exposure notifications during smartphone encounters are generated through a central authority (eg, a hospital) and enough of them are generated in batches, for future use and for constructing contact graphs of users when they are infected [136]. A DoS on this server could prevent the IDs and relevant estimations to reach the targets, and the affected persons would have a false sense of safety since they are no longer notified about encountered contacts. In any case, the effect of DDoS is likely higher in centralized and hybrid P2P PHS than in decentralized P2P PHSs such as P2P IHE [6,51]. This is because of the presence of single points that manage other users in the network. However, centralized control mechanisms also ease the tracing of attackers and reduce the probability of DDoS attacks.

#### P2P Traffic Blockade

In 2008, P2P networks accounted for almost 53% of internet traffic in Germany, followed by web browsing (26%) and streaming (7%) [122]. With respect to P2P network traffic, BitTorrent accounted for 37%, web browsing for 15%, and eDonkey for 13% of P2P internet traffic [122]. Given the high proportion of P2P traffic in most regions, it is not surprising that a number of internet service providers (ISPs) are using advanced filtering techniques to impose bandwidth limits and throttle or block traffic associated with P2P systems, for instance, by using port numbers, flow features, and deep packet inspections [46,100,121]. In 2012, the United Kingdom High Court ordered, for example, some ISPs (eg, O2, Virgin Media, and TalkTalk) to block BitTorrent P2P traffic owing to its potential for copyright infringements [120].

#### Impact of P2P Traffic Blockade on P2P PHSs

The consequence of a P2P traffic blockade on any type of P2P PHS could be high because the effect could render the system unavailable over the network, for instance, in a situation where ISPs realize a high proportion of internet traffic caused by P2P networks and impose bandwidth limits or block the traffic. If any P2P PHS user is affected by the blockage, P2P PHSs, for instance, for remote sharing of medical records or COVID-19 exposure notifications will be disrupted. This can potentially affect patient health and contribute to virus spread. As a workaround, users can move to a different region that does not block traffic because P2P systems are not bound by borders. The chances of being affected by a P2P traffic blockade when using a PHS is higher in regions that often use network traffic blockades as a public policy instrument (eg, in authoritarian regimes).

## Discussion

### Principal Findings

Our findings support the idea that P2P system security is a process rather than a product [33]. Moreover, security encompasses not only technical issues but also human and management problems. Therefore, it is highly relevant for the development and use of P2P PHSs to consider the security issues in P2P networks and the techniques used to exploit them, the security requirements to prevent attacks, peculiarities of attacks, and potential attacker profiles. Our findings are presented in Tables 3 and 4. Security issues such as malware, social engineering attacks, eclipse attacks, DDoS attacks, pollution attacks, and P2P traffic blockades pose high threats (in case of a successful attack) and have a high probability of being exploited in P2P PHSs owing to the high number of factors contributing to their chances of successful exploitation (Table 3); moreover, they can put any P2P PHS out of service, which can potentially affect patients’ state of health. For illustrative purposes, we discuss the factors and scores for malware and eclipse attacks in detail below (refer to the section *Identified Security Issues and Their Impact on P2P PHSs* for a detailed discussion of the security issues).

The effect of any malware type depends on its propagation speed and power. Malware that compromised a PHS can be inadvertently spread by the patient (eg, when it is hidden in a patient’s health records). Other factors promoting security issues in P2P networks (set-and-forget, no borders, digital winds spreading files, growing use, and network heterogeneity) and no content verification (Table 3) can fuel malware propagation in the network. If attackers compromise super nodes (eg, practitioners or hospital nodes), they can spread malware even more easily. A successful malware attack (eg, Antinny [85,86] or Code-Red [78]) on any P2P PHS can affect the CIA properties of the network and may cause a delay in treatment or even loss of life (eg, the case of a patient who died after a malware hit a hospital in Germany [130]). Malware can attack various network layers (user, network, or transport layers) to mount DoS attacks, poison the network, block P2P traffic, or compromise users’ identities or health data.

The severity of malware is low in centralized P2P PHSs (eg, the e-toile framework in Switzerland [21] or P2P PHR [6]; Table 4) because the central index server can simply be used as a trusted computing base [25,26] or a point to deploy control measures to mitigate the propagation of malware in the network. The severity of malware is medium in hybrid P2P PHSs (eg, P2HR [20]), our proposed P2P PHS architecture (Figure 3; Table 4), because there are no central attack profiles, and a federated data ecosystem multiplies the effort required for malware attacks by the number of federated subnetworks. The severity of malware is high in decentralized P2P PHSs (eg, P2P IHE [51]; Table 4) because of the lack of a trusted computing base and high responsibility for individual users to maintain routing information (DHT networks) and security measures [25,26]. Once the neighbor lists of users are infected by malware, the malware can spread further (eg, using a PRS strategy) through the nodes’ subnetworks, which contributes to the malware's high propagation speed [78,82].

Factors such as use and network heterogeneity, no borders, and snooping nodes promote the impact of eclipse attacks on P2P networks (Table 3). In most cases, a successful eclipse attack allows an attacker to eavesdrop on the conversation between peers in the network without potentially compromising the victim's system. This impacts the reliability, autonomy, connectivity, and CIA properties of P2P networks [72,106,114].

The severity of eclipse attacks is low in centralized P2P PHS (eg, the e-toile framework in Switzerland [21]; Table 4) because of the difficulty for users to create multiple fake identities (as required to mount an eclipse attack [54,77,81]) and the likely presence of trusted computing infrastructure in centralized P2P PHSs. Nevertheless, attacks on central index servers (or super peers in hybrid P2P PHSs) are likely to be able to snoop network communications. The severity of eclipse attacks is medium in hybrid and decentralized P2P PHSs (eg, P2P IHE [51]) or our proposed P2P PHS architecture [Figure 3; Table 4]), as eclipse attacks require a high number of compromised nodes and are usually achieved through attacks on routing mechanisms [47,54,77,81]. Decentralized and hybrid P2P PHSs require routing mechanisms (eg, DHT) to facilitate health information exchange and communication between patients and practitioners.

**Table 3:** Factors promoting the security issues.

Security issues	Factors promoting the security issues						
	Inadvertent sensitive information disclosure	Set-and-forget	No borders	Digital winds Spreading Files	Use and network heterogeneity	No content verification	Snooping nodes
Malware	✓^a^	✓	✓	✓	✓	✓	✓
Social engineering attack	✓		✓	✓	✓	✓	✓
Poisoning the network			✓	✓	✓	✓	✓
Sybil attack			✓		✓		✓
Eclipse attack			✓		✓		✓
DDoS attack		✓	✓		✓	✓	✓
Pollution	✓	✓	✓	✓	✓	✓	✓
P2P^b^ traffic blockade					✓	✓	✓

^a^Factor present.

^b^P2P: peer-to-peer.

**Table 4:** Severity ratings for peer-to-peer patient-centered health care information system security.

Security issues	Severity score on P2P PHS^a^			Exemplary security measures
	Centralized	Hybrid	Decentralized	
Malware	Low	Medium	High	Firewall Antivirus and antispyware Mobile agent–based intrusion detection system Access policies
Social engineering attack	Medium	Medium	Medium	Education and awareness training
Poisoning the network	Low	Medium	High	Authentication protocol Trust and reputation system Access policies
Sybil attack	Low	Low	Medium	Authentication protocol Trust and reputation system End-to-end encryption
Eclipse attack	Low	Medium	Medium	Authentication protocol Trust and reputation system End-to-end encryption Access policies
DDoS^b^ attack	High	Medium	Medium	Firewall Mobile agent–based intrusion detection system Bandwidth limitation per node Access policies
Pollution	Low	Medium	Medium	File and content verification Trust and reputation system End-to-end encryption Removal of polluted content
P2P traffic blockade	High	Medium	Low	End-to-end encryption Encryption of P2P traffic

^a^P2P PHS: peer-to-peer patient-centered health care information system.

^b^DDoS: distributed denial-of-service.

### Protecting P2P PHSs Against Security Issues

Under normal circumstances, patient-physician relationships are based on trust, and P2P systems generally require trust between their participants [46]. However, uncertainties regarding the protection of user data, single points of failure, and the integrity of the super peers remain. Under our proposed PHS architecture (Figure 3), a trusted registration authority (eg, the German HTI or a hospital) is introduced to the network to handle administrative tasks such as authentication and verification and can also issue or revoke credentials to users based on their behavior [30]. End-to-end encryption [137] can be used to maintain confidentiality in health care information systems [30] and to reduce the trust required for other network participants. For instance, the state-of-the-art cryptographic protocol Signal for end-to-end encryption, which is used by popular instant messaging apps [138], including WhatsApp, Wire, and Facebook Messenger, can be used. Security analyses of the Signal protocol show that it can resist most known attacks [139]. Furthermore, transparency mechanisms can be used to make it easier to hold a provider accountable for violating users’ trust [26], for example, certificate transparency can be managed by a set of services and neutral auditors to keep track of X.509 certificates of providers and quickly observe rogue or hacked certificate authorities. Such security techniques reduce the impact of eclipse attacks, DDoS attacks, pollution attacks, poisoning attacks, and P2P traffic blockade on P2P networks [52,81]. For example, an intercepted message can be rendered useless for eclipse attackers by encrypting it.

**Figure 9:**
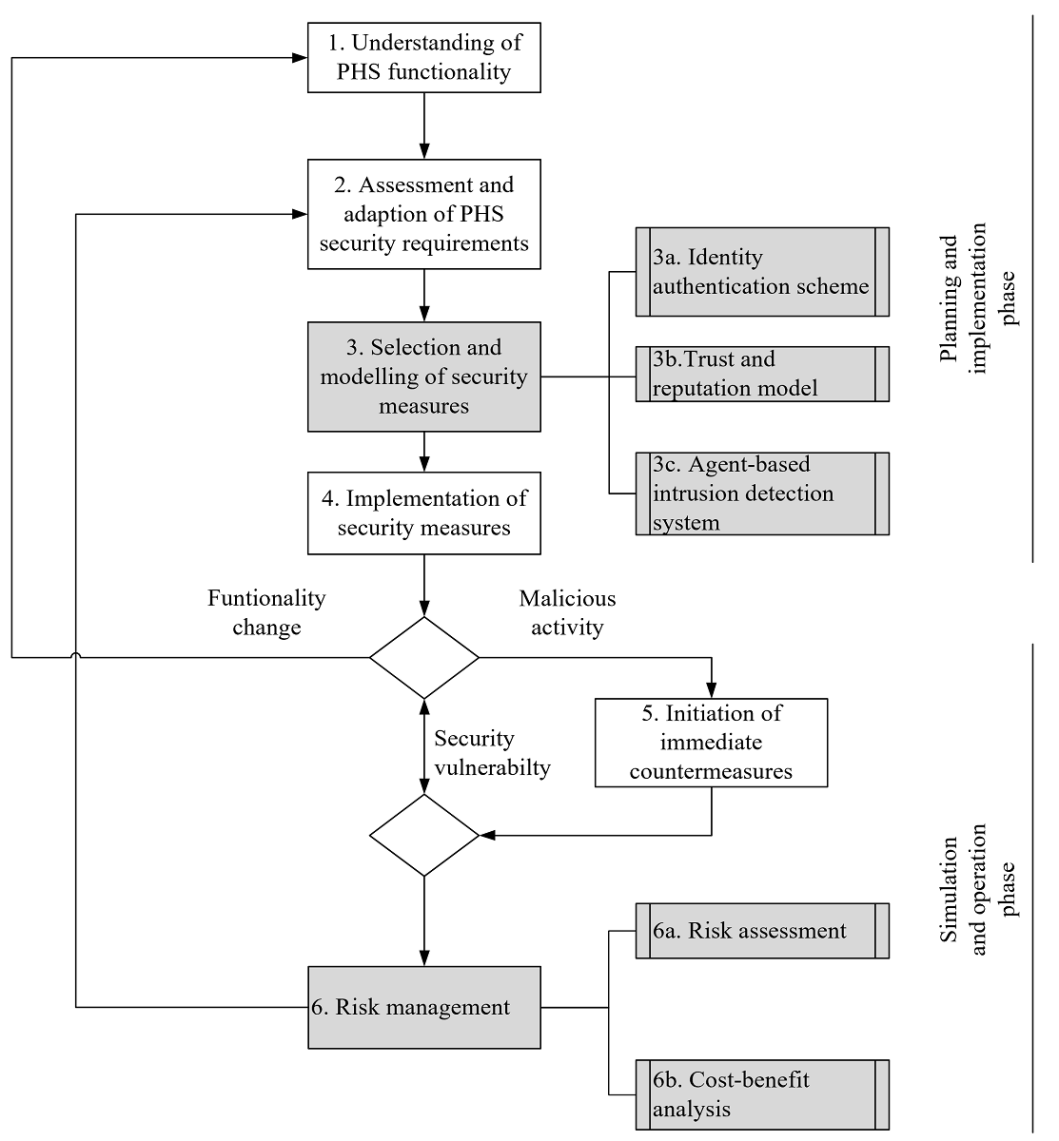
Guidelines for provision of the patient-centered health care information system (PHS) while ensuring security.

A discussion of all possible security measures (see Table 4 for examples) for each identified security issue is beyond the scope of this study. In line with the identified security issues, we focus on trust and reputation models (TRM), identity authentication schemes (IAS), and agent-based intrusion detection systems (IDSs). As an overarching guideline, we extended an extant guideline for secure provision of PHSs [2] (Figure 9) with 2 additional steps (*selection and modeling of security measures* [step 3] and *risk assessment* [step 6]). The guideline is useful for supporting individual PHS providers to deal with the complexity of securing P2P PHSs.

An effective IAS addresses security issues such as Sybil attacks, poisoning attacks, pollution attacks, and MitM attacks [65,81,140] and is essential for health care information systems [2,30]. By authenticating users and resources shared, the origin of pollution or poisoning attacks can be traced, and the attackers can be held accountable. Individual PHS providers leveraging an effective IAS can strengthen security, which has the potential to increase patients’ intention to use P2P PHSs. In Germany, the German HTI planned to provide user authentication through smart cards as a security measure for PHS providers [65,141]. However, the introduction of national HTIs often leads to ambiguous, expensive, and protracted projects [65,141]. Until such solutions are widely available, developers of P2P PHSs should consider the use of other IASs for the secure provision of PHSs in public networks [65].

Reputation systems are used to determine the trustworthiness of nodes and to mitigate Sybil, poisoning, pollution, and MitM attacks [142]. Reputation management for resources being shared and peers [143] reduces vulnerabilities such as ID stealth or pseudospoofing [144,145]. TRM techniques can be leveraged in P2P PHS in any situation where a party misbehaves (eg, by supplying inappropriate data to a PHS). Patients can report misbehavior to reputation systems so that it can be reflected in the reputation of the misbehaving party. Polluted resources can also be reported and removed if their reputation is too low [72,73,75,81].

To address the issues of malicious peers, worms, and DDoS attacks in the network, an intelligent mobile agent–based IDS can be deployed in strategic locations (eg, at a hospital node in our proposed P2P PHS architecture, Figure 3; in the DHT network in decentralized P2P PHSs such as P2P IHE [6,51]; or at central index servers of centralized PHSs such as the e-toile PHS [21]) to protect the corresponding subnetworks in P2P networks. There are prototypes of scalable and decentralized agent-based IDS that use 3 types of algorithms (heavy, medium, and light scan algorithms) to detect malicious activities as early as possible [87,146,147]. Backpropagation neural network techniques can be used in IDS to reduce the response times and false alarm rates [148,149]. To improve detection latency and load balancing, a collaborative IDS uses publish and subscribe techniques to selectively route evidence of malicious activities between peers in the network using distributed lookup mechanisms [150,151]. Worms scan and infect certain ports in a network. A firewall can be used to monitor, filter, block, and blacklist them; antivirus and antispyware tools can be leveraged to remove or quarantine any suspicious file [81]. The DDoS can be mitigated by limiting the download bandwidth for each node. Other policies, such as restricting P2P access to verified directories and scanning each file before opening, can mitigate the impact of DDoS, malware, and poisoning attacks [97].

We added risk management (step 6) to the guideline for secure provision of PHSs (Figure 9) to allow for prioritization of security issues with higher impact and for the efficient use of available resources [152]. Risk assessment (step 6a) focuses on the identification and assessment of security issues based on the likelihood of occurrence and the severity of exploits. The cost-benefit analysis involves an analysis of the costs associated with recovering from security breaches. In a situation where the costs for mitigation are higher than the potential impact of a security issue, P2P PHS providers may choose to accept some level of risk.

### Limitations

This research focuses on security engineering for P2P PHSs. Legal issues with respect to health care security management are beyond the scope of this study. A further limitation of this study is that P2P PHS is an emerging phenomenon; therefore, our study does not provide real-world experiments or a review of past P2P PHS security incidents. Moreover, the bandwidth, computation, and storage cost analyses of the proposed P2P infrastructure, how usability and deployability will affect P2P PHS adoption, and how to handle patient registration with multiple PHS providers are beyond the scope of this study.

### Contributions

Our research provides a foundation for understanding P2P system architectures and their advantages and disadvantages. We propose and discuss a federated architecture (Figure 3) suitable for PHS deployment, which could be adopted by any P2P PHS provider, such as insurance companies, hospitals, or other parties who want to implement P2P PHSs while maintaining security. On the basis of the 3 different archetypical P2P system architectures, we elicited and reviewed the inherent security issues and factors promoting the security issues (Table 3). Moreover, we discuss the consequences of the security issues and apply a severity scoring system (Table 4), signifying the impact of each security issue for the 3 different architectures of P2P PHSs—centralized, hybrid, and decentralized—based on the CVSS definitions (Multimedia Appendix 2). Although a comprehensive discussion of security measures to address each identified security issue is beyond the scope of this study, we offer an overview of potential security measures that are useful for maintaining security in P2P PHSs. We also extended a guideline for the secure provision of PHSs in public networks (Figure 9) for the P2P PHS context [2].

P2P PHSs (eg, COVID-19 contact tracing systems such as PEPP-PT [22] or OnePatient [15]) require research from many perspectives to facilitate widespread use because they are an emerging phenomenon, pose major security issues (eg, by requiring patients to manage information security largely by themselves [65]), and are understudied. Extant research on PHS security, privacy, and end-user features [2,28-31] focuses on centralized and DLT-based PHS. Our research serves as an introduction to P2P PHSs and potential security issues and countermeasures. From an ethical perspective, our study is of interest to initiatives aimed at empowering patients to take ownership of and control access to their health data. P2P PHSs promote socially desirable design features such as openness, reduced dependence on platforms, abandonment of data silos, and secure patient-to-practitioner communication. Given that the security challenges are appropriately addressed, P2P PHSs are also promising for simplifying the implementation of data protection principles (eg, GDPR [8,34]). Secure P2P PHSs will not only attract more stakeholders but will also be more efficient in achieving the goals of patient-centered digital ecosystems [153].

### Future Research

Opportunities for future research include improved designs of security models, such as IAS, TRM, and intelligent mobile agent–based IDS, to strengthen security. PHSs have other more safety-related security requirements that should also be incorporated in their design, such as emergency access and guardian support. Such features are vital for P2P PHS to facilitate access in situations where patients are incapacitated. However, they are also likely to invoke privacy concerns and data protection challenges, as they require access to sensitive information without consulting patients. By using reliable and patient-centered backup options, P2P PHS providers can integrate identity authentication management in backup servers to facilitate the replacement of patient credentials in a situation where they lose access to their credentials (eg, a stolen laptop). In addition to the development of approaches to improve education and awareness of patients regarding information security challenges inherent to the sharing of data with third parties [8], a questionnaire-based study focusing on other P2P PHS stakeholders and asking about their security and privacy concerns with respect to P2P PHSs could yield valuable contributions. A guideline for the evaluation of P2P PHSs based on information security standards (eg, ISO 27799:2016) could also be very useful.

### Conclusions

The idea of P2P PHSs to break up barriers among patients, health care systems, physicians, and other stakeholders is appealing. From the patients’ perspective, being empowered to conveniently take ownership of and control access to their health data through PHS might bring forth a digital ecosystem that makes patients a more active contributor in their own care and can streamline health care activities such as receiving and accurately interpreting laboratory test results. In the United States, HIPAA [6] specifies that patients have the liberty “to see and get copies of their records, and request amendments”; however, the act does not go into detail on appropriate approaches to give access [3,30,154]. Currently, PHSs use DLT, P2P technology, or centralized databases for deployment. To mitigate the impact of security issues in centralized databases and the lack of fit of DLT with PHS use cases, P2P PHSs emerged (eg, OnePatient [15], doc.ai brands [7], or COVID-19 proximity tracing systems such as Stoop [24]), which store health records locally (on any patient edge device such as a mobile phone, a tablet computer, a desktop computer, etc) under the control of individual device owners.

The benefits of P2P networks for PHSs include more options for privacy self-management, autonomous control of infrastructure, and high availability. However, these advantages are associated with complications, as patients must also manage information security largely by themselves. Gartner claims that costs for remediating security issues would be reduced by 75% if only 50% of system vulnerabilities were detected and remediated before production [155]. Building a successful P2P system that does not result in privacy or security violations for users is difficult [26] and entails a collective effort that fixes the remaining problems (eg, absence of a centralized entity to detect malicious attacks and increased chances of exposing network traffic patterns) with clear considerations of network security and ease of use.

The enormous value of health data requires the provision of security measures to protect PHSs from cyberattacks. Overcoming security and privacy barriers in P2P PHS is also important for increasing patients’ intention to use PHSs. PHS providers and developers should neither ignore the inherent or past security issues of P2P systems nor be careless about future ones.

## Appendix

Multimedia Appendix 1 List of individual journals and conferences.

Multimedia Appendix 2 Definition of Consequence of Exploitation. The rate estimation was guided by the Common Vulnerability Scoring System which provides a way to capture the principal characteristics of a vulnerability and produce a numerical score reflecting its severity.

## Abbreviations

CIA: confidentiality, integrity, availability
CVSS: Common Vulnerability Scoring System
DDoS: distributed denial-of-service
DHT: Distributed Hash Tables
DLT: distributed ledger technology
DoS: denial-of-service
GDPR: General Data Protection Regulation
HIPAA: Health Insurance Portability and Accountability Act
HTI: health care technology infrastructure
IAS: identity authentication schemes
IDS: intrusion detection systems
IHE: integrating health care enterprise
ISP: internet service provider
MitM: man-in-the-middle
P2P: peer-to-peer
PEPP-PT: Pan-European Privacy-Preserving-Proximity-Tracing
PHS: patient-centered health care information system
PRS: pure random scan
TCP: transmission control protocol
TRM: trust and reputation model
