# Supplementary material for: Security Engineering of Patient-Centered Health Care Information Systems in Peer-to-Peer Environments: Systematic Review
Source: J Med Internet Res. 2021 Nov 15;23(11):e24460. doi: 10.2196/24460 (PMC8663665; doi:10.2196/24460)
Supplement: Multimedia Appendix 1 [file jmir_v23i11e24460_app1.docx]

# Multimedia Appendix 1

List of Individual Journals and Conferences

| Database | S/N | Source | Number of Studies Selected |
| --- | --- | --- | --- |
| PubMed | 1 | Journal of Medical Internet Research | 1 |
|  | 2 | Sensors | 1 |
| ACM | 3 | IWQoS '11: Proceedings of the Nineteenth International Workshop on Quality of Service | 1 |
|  | 4 | [ICDT '03: Proceedings of the 9th International Conference on Database Theory](https://dl.acm.org/doi/proceedings/10.5555/645505) | 1 |
|  | 5 | [InfoScale '06: Proceedings of the 1st international conference on Scalable information systems](https://dl.acm.org/doi/proceedings/10.1145/1146847) | 1 |
| Science Direct | 6 | Journal of Procedia Computer Science | 1 |
|  | 7 | Journal of Procedia Technology | 1 |
|  | 8 | Journal of Computer Communications | 1 |
| Scopus | 9 | Mobile and Wireless Networks Security - Proceedings of the MWNS 2008 Workshop | 1 |
|  | 10 | Proceedings of the National Conference on Artificial Intelligence | 1 |
|  | 11 | 8th International IFIP-TC 6 Networking Conference, NETWORKING 2009 | 1 |
|  | 12 | International Conference for Internet Technology and Secured Transactions, ICITST 2009 | 1 |
|  | 13 | IEEE Journal on Selected Areas in Communications | 1 |
|  | 14 | Intelligent Systems Reference Library | 1 |
|  | 15 | Proceedings of the 24th USENIX Security Symposium | 1 |
|  | 16 | Proceedings of the 2015 IFIP/IEEE International Symposium on Integrated Network Management, IM 2015 | 1 |
|  | 17 | Proceedings - IEEE Symposium on Security and Privacy | 1 |
| Springer | 18 | iNetSec 2009 - Open Research Problems in Network Security | 1 |
|  | 19 | ISSS 2012 - International Symposium on Software Security | 1 |
|  | 20 | International Conference on Database Theory - ICDT 2003 | 1 |
|  | 21 | International Conference on Embedded and Ubiquitous Computing  EUC 2006: Emerging Directions in Embedded and Ubiquitous Computing | 1 |
|  | 22 | Peer-to-Peer Systems and Applications | 1 |
|  | 23 | Peer-to-Peer Networking and Applications | 1 |
| IEEE | 24 | 2009 11th International Conference on Advanced Communication Technology | 1 |
|  | 25 | 2012 14th International Conference on Advanced Communication Technology (ICACT) | 1 |
|  | 26 | 2013 8th International Conference on Communications and Networking in China (CHINACOM) | 1 |
|  | 27 | 2011 International Conference on Cloud and Service Computing | 1 |
|  | 28 | 2009 Second International Conference on the Applications of Digital Information and Web Technologies | 1 |
|  | 29 | 2012 International Conference on Cyber-Enabled Distributed Computing and Knowledge Discovery | 1 |
|  | 30 | 2010 2nd International Workshop on Intelligent Systems and Applications | 1 |
|  | 31 | 2013 IEEE International Conference on Communications (ICC) | 1 |
|  | 32 | 2008 International Symposium on Electronic Commerce and Security | 1 |
|  | 33 | 2010 IEEE/ACM Int'l Conference on Green Computing and Communications & Int'l Conference on Cyber, Physical and Social Computing | 1 |
|  | 34 | IEEE Communications Surveys & Tutorials | 1 |
|  | 35 | 2009 International Conference on Computational Intelligence and Natural Computing | 1 |
|  | 36 | 2011 IEEE Conference on Computer Communications Workshops (INFOCOM WKSHPS) | 1 |
|  | 37 | 2012 8th International Conference on Wireless Communications, Networking and Mobile Computing | 1 |
|  | 38 | 2012 IEEE International Conference on Pervasive Computing and Communications Workshops | 1 |
|  | 39 | Proceedings of the 41st Annual Hawaii International Conference on System Sciences (HICSS 2008) | 1 |
|  | 40 | 2012 8th International Conference on Computing and Networking Technology (INC, ICCIS and ICMIC) | 1 |
| Semantic Scholar | 41 | International Journal of Applied Engineering Research | 1 |
|  | 42 | IOSR Journal of Electrical and Electronics Engineering (IOSR-JEEE) | 1 |
|  | 43 | Proceedings of the 2017 ACM SIGSAC Conference on Computer and Communications Security | 1 |
|  | 44 | Proceedings on Privacy Enhancing Technologies | 1 |
|  | 45 | Cornell University/arXiv.org | 1 |
|  | 46 | WorldComp Proceedings | 1 |
|  | 47 | Media Net Lab: Kent State University, Networking and Media Communication | 1 |
|  | 48 | citeseerx.ist.psu.edu | 1 |
|  | 49 | International Journal on Cybernetics & Informatics (IJCI) | 1 |
