# Supplementary material for: Security Engineering of Patient-Centered Health Care Information Systems in Peer-to-Peer Environments: Systematic Review
Source: J Med Internet Res. 2021 Nov 15;23(11):e24460. doi: 10.2196/24460 (PMC8663665; doi:10.2196/24460)
Supplement: Multimedia Appendix 2 [file jmir_v23i11e24460_app2.docx]

# Multimedia Appendix 2

Definition of Consequence of Exploitation. The rate estimation was guided by CVSS which provides a way to capture the principal characteristics of a vulnerability and produce a numerical score reflecting its severity.

| Definition | Explanation |
| --- | --- |
|  |  |
| High | Confidentiality—there is a very high probability for total information loss, resulting in all system files being revealed  Integrity—there is a total compromise of system integrity. There is a complete loss of system protection, resulting in the entire system being compromised  Availability—there is a total shutdown of the affected resource. The attacker can render the resource completely unavailable |
| Medium | If any of the two CIA statements is compromised. For instance:  Confidentiality—there is considerable informational disclosure  Integrity—a modification of some system files or information is possible, but the attacker does not have control over what can be modified, or the scope of what the attacker can affect is limited  Availability—there is reduced performance or interruptions in resource availability |
| Low | If one of the CIA statements is compromised. For instance:  Confidentiality—there is considerable informational disclosure  Integrity—there is low impact to the integrity of the system  Availability—there is no impact on the availability of the system. |
